# The β-arrestin1/endothelin axis bolsters ovarian fibroblast-dependent invadosome activity and cancer cell metastatic potential

**DOI:** 10.1038/s41419-024-06730-6

**Published:** 2024-05-22

**Authors:** Danila Del Rio, Ilenia Masi, Valentina Caprara, Flavia Ottavi, Gabriele Albertini Petroni, Erica Salvati, Daniela Trisciuoglio, Sara Maria Giannitelli, Anna Bagnato, Emanuele Mauri, Francesca Spadaro, Laura Rosanò

**Affiliations:** 1 Institute of Molecular Biology and Pathology (IBPM), National Research Council (CNR), Rome, 00185 Italy; 2 Unit of Preclinical Models and New Therapeutic Agents, IRCCS-Regina Elena National Cancer Institute, Rome, 00144 Italy; 3 Department of Science and Technology for Sustainable Development and One Health, University Campus Bio-Medico di Roma, Rome, 00128 Italy; 4 Department of Chemistry Materials and Chemical Engineering, University Politecnico di Milano, 20133 Milano, Italy; 5 Confocal Microscopy Unit, Core Facilities, Istituto Superiore di Sanità, Rome, 00161 Italy

**Keywords:** Gynaecological cancer, Cell migration

## Abstract

Recruitment of fibroblasts to tumors and their activation into cancer-associated fibroblasts (CAFs) is a strategy used by tumor cells to direct extracellular matrix (ECM) remodeling, invasion, and metastasis, highlighting the need to investigate the molecular mechanisms driving CAF function. Endothelin-1 (ET-1) regulates the communication between cancer and stroma and facilitates the progression of serous ovarian cancer (SOC). By binding to Endothelin A (ET_A_) and B (ET_B_) receptors, ET-1 enables the recruitment of β-arrestin1 (β-arr1) and the formation of signaling complexes that coordinate tumor progression. However, how ET-1 receptors might “educate” human ovarian fibroblasts (HOFs) to produce altered ECM and promote metastasis remains to be elucidated. This study identifies ET-1 as a pivotal factor in the activation of CAFs capable of proteolytic ECM remodeling and the generation of heterotypic spheroids containing cancer cells with a propensity to metastasize. An autocrine/paracrine ET-1/ET_A/B_R/β-arr1 loop enhances HOF proliferation, upregulates CAF marker expression, secretes pro-inflammatory cytokines, and increases collagen contractility, and cell motility. Furthermore, ET-1 facilitates ECM remodeling by promoting the lytic activity of invadosome and activation of integrin β1. In addition, ET-1 signaling supports the formation of heterotypic HOF/SOC spheroids with enhanced ability to migrate through the mesothelial monolayer, and invade, representing metastatic units. The blockade of ET_A/B_R or β-arr1 silencing prevents CAF activation, invadosome function, mesothelial clearance, and the invasive ability of heterotypic spheroids. In vivo, therapeutic inhibition of ET_A/B_R using bosentan (BOS) significantly reduces the metastatic potential of combined HOFs/SOC cells, associated with enhanced apoptotic effects on tumor cells and stromal components. These findings support a model in which ET-1/β-arr1 reinforces tumor/stroma interaction through CAF activation and fosters the survival and metastatic properties of SOC cells, which could be counteracted by ET_A/B_R antagonists.

## Introduction

The supporting role of stromal cells in the metastatic ability of serous ovarian cancer (SOC) cells has been extensively demonstrated, highlighting how the co-evolution of tumor cells with fibroblasts in the tumor bulk strongly influences the metastatic journey and response to therapy [1–3]. Fibroblasts in the tumor microenvironment (TME), also referred to as cancer-associated fibroblasts (CAFs), are a highly heterogeneous cellular component originating from different precursors, including resident tissue fibroblasts, and mesothelial cells (MCs). CAFs account for several malignant properties, such as invasion and metastasis, via cytokine/chemokine secretion, and extracellular matrix (ECM) remodeling, capable of impacting the clinical outcomes of SOC patients [4–6]. Different omics studies revealed an ecosystemic landscape of high-grade (HG)-SOC at early or late stages linked to heterogeneity within the TME, where the most aggressive “fibrosis” or “mesenchymal” CAF subtype is associated with poor patient survival. Moreover, the high content of CAFs and a subtype of CAFs expressing the EMT features is enriched in metastatic tumors. In addition, several signaling pathways, ligands, and receptors are involved in the communication between CAFs and cancer cells with prognostic and therapeutic relevance [7–12]. An abundance of CAFs is associated with advanced stage and metastasis to the omentum and lymph nodes, contributing to the formation of the premetastatic niche and the failure of drug treatments by exchanging signals with tumor cells [7, 13]. CAFs can form the core of spheroids and serve as a scaffolding to aggregate in heterotypic spheroids with cancer cells, via the activation of cadherins and integrins, representing metastatic units with high malignant potential. These heterotypic spheroids contribute to peritoneal dissemination given the high degree of invasiveness, enhanced pro-survival signaling, and adhesion to mesothelium [14]. In addition, fibroblasts are characterized by extensive matrix-synthesizing and matrix-remodeling capacities, pivotal for establishing an invasion-permissive TME [15, 16]. In this context, Src-transformed fibroblasts or fibroblasts activated by growth factors, signaling pathways, and environmental cues, might form invadosomes, integrin-based matrix adhesion, composed of actin regulators, as TKS5 and cortactin, facilitating matrix degradation, in different human diseases including cancers [17–21].

In SOC, mediators present in ascites initiate or perpetuate CAF activation [2]. Since the available treatments for this tumor remain limited, the knowledge of the interaction between CAFs and tumor cells via soluble mediators is essential for the development of effective treatment strategies.

Within the SOC-associated tumor-promoting factors, the peptide endothelin-1 (ET-1) and its interactions with the cognate G-protein coupled receptors (GPCRs), Endothelin A (ET_A_R), and B (ET_B_R) receptors, support tumor progression, activating key pathways in invasion and metastasis, through the differential cooperation of β-arrestins (β-arrs) with proteins in cytosolic and nuclear compartments [22, 23]. Although the predominant effects have been studied on cancer cells, the ET-1 axis is involved in regulating the activities of cancer-associated stromal cells, including those related to ovarian TME, such as endothelial and lymphatic cells, and MCs [24–28]. The ET_A_R/β-arr1 signaling promotes invadopodia-dependent invasion [29]. Moreover, ET_A_R/β-arr1 drives integrin α5β1 (intα5β1) inside-out activation and promotes the survival of 3D spheroids, with mesothelium-intercalation capacity and invasive behavior [30]. However, the role of ET-1/β-arr1 in ovarian fibroblast activation and ECM remodeling is completely unknown. Additionally, it has been reported that β-arr1 enhances fibroblast migration when co-cultured with cancer cells by modulating cofilin activity [31], suggesting a new uncovered ET-1/β-arr1-driven pathway in CAFs. Here, we studied how the ET-1/β-arr1 axis promotes ovarian CAF behavior and ECM remodeling, supporting tumor/stroma communication and the favorable behavior in the SOC premetastatic niche, and tested the therapeutic effects of inhibiting this axis targeting fibroblast/cancer cell metastatic units.

## Results

### ET-1/β-arr1 axis promotes ovarian fibroblast activation

**Fig. 1:**
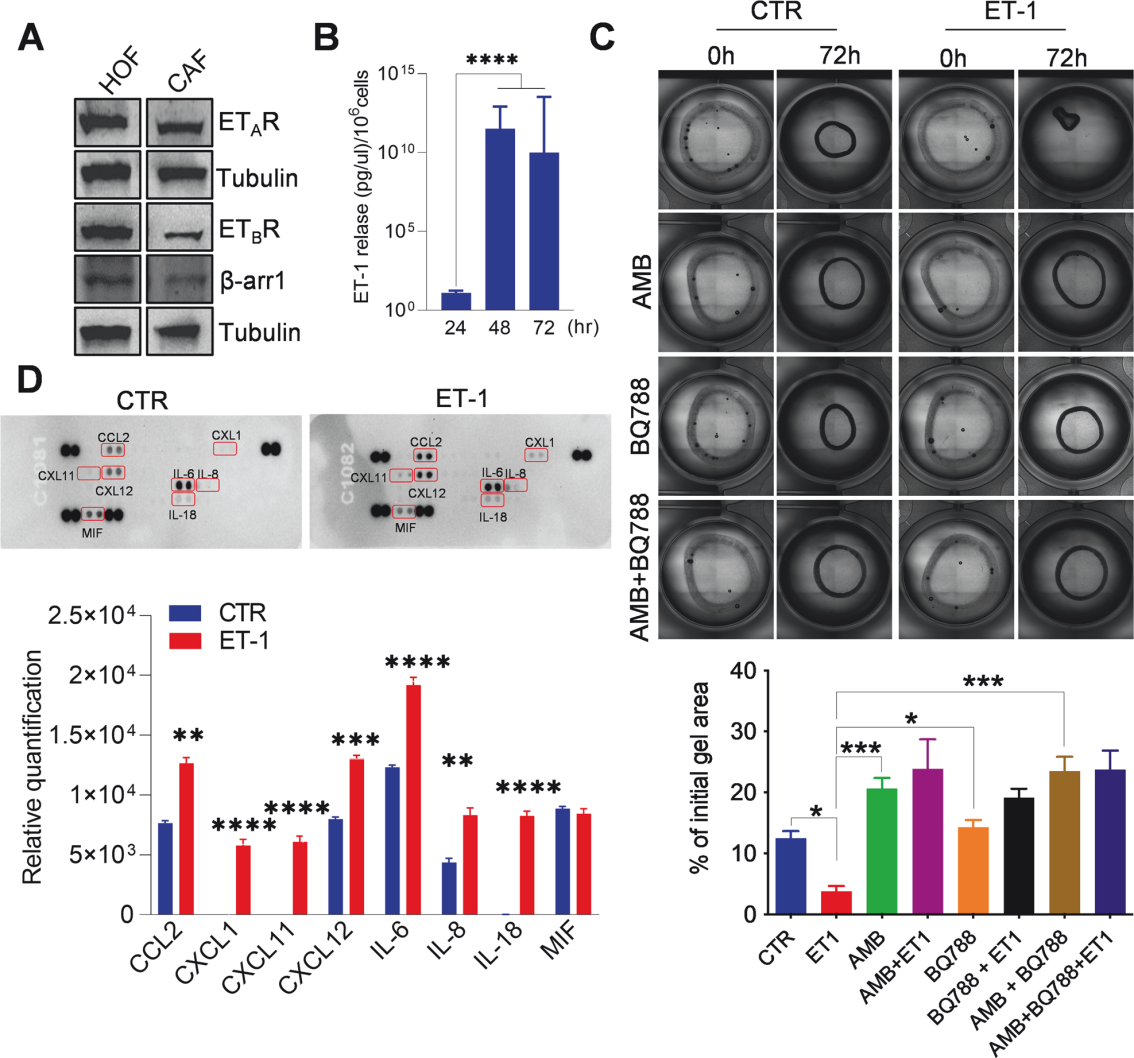
ET-1/β-arr1 axis activates human ovarian fibroblasts (HOFs). **A** Representative Western blotting (WB) of whole cell lysates probed with Abs to ET_A_R, ET_B_R, and β-arr1. Tubulin, loading control. **B** ET-1 secretion measured by ELISA in conditioned media (CM) from HOFs at indicated time points. Histograms represent the mean ± SD. Statistics were obtained using One-way ANOVA, *n* = 2. **C** Collagen contraction assay by using HOFs stimulated with ET-1 and/or AMB (ET_A_R antagonist) and/or BQ788 (ET_B_R antagonist) after 72 h. Histograms represent the mean ± SD; statistics were obtained using One-way ANOVA. **D** Cytokine release evaluated in CM from untreated or ET-1-treated HOFs (24 h) by using cytokine profiler array. Histograms represent the mean ± SD, n = 2 of the analytes detected; statistics were obtained using One-way ANOVA.

To investigate ET-1 as a candidate soluble mediator affecting CAF behavior and the involvement of β-arr1, we used human primary fibroblasts derived from the normal ovary (HOFs), characterized by the lower basal expression of fibroblast activation markers, such as α-SMA, vimentin, confirming their inactivated phenotype, compared to ovarian cancer-associated fibroblasts (CAFs) (Supplementary Fig. 1A). Both HOFs and CAFs express ET-1, ET_A_R, and ET_B_R, as well as β-arr1 (Fig. 1A, and Supplementary Fig. 1B). Moreover, HOFs secrete ET-1 in their conditioned media (CM) reaching the level of 11 pg/μl/10^6^ cells (Fig. 1B).

In agreement with previous data [26–28, 32], ET-1 induces HOFs to acquire features of CAFs, as an increase in platelet-derived growth factor receptor (PDGFR), fibroblast activated protein (FAP), and vimentin expression (Supplementary Fig. 1C). In addition, the treatment with ET-1 enhances the expression of ET_B_R and β-arr1 and decreases that of ET_A_R (Supplementary Fig. 1D), suggesting that autocrine or paracrine ET-1 secretion might regulate ET-1/β-arr1 axis. Moreover, an enhanced collagen contraction is evident upon ET-1 addition (Fig. 1C). To analyze the involvement of specific receptors in these effects, we added either the ET_A_R antagonist Ambrisentan (AMB), the ET_B_R antagonist BQ788, or both. The addition of AMB, BQ788, or their combination, blocks the ET-1 effect, shrinking the gels to the HOF control level (Fig. 1C). Since CAFs secrete soluble factors to reconstruct the TME and promote cancer cell invasion and metastasis [17], we used cytokines profiler arrays to analyze CMs from HOFs unstimulated or stimulated with ET-1. Enhanced levels of many cytokines related to cancer invasion and migration are found in the CMs of ET-1-treated cells, including IL-6, IL-8, CXCL11, and CXCL12 (Fig. 1D), further supporting the hypothesis that ET-1 is a driver of activated fibroblasts.

### ET-1 regulates invadosome activation and Intβ1 signaling

**Fig. 2:**
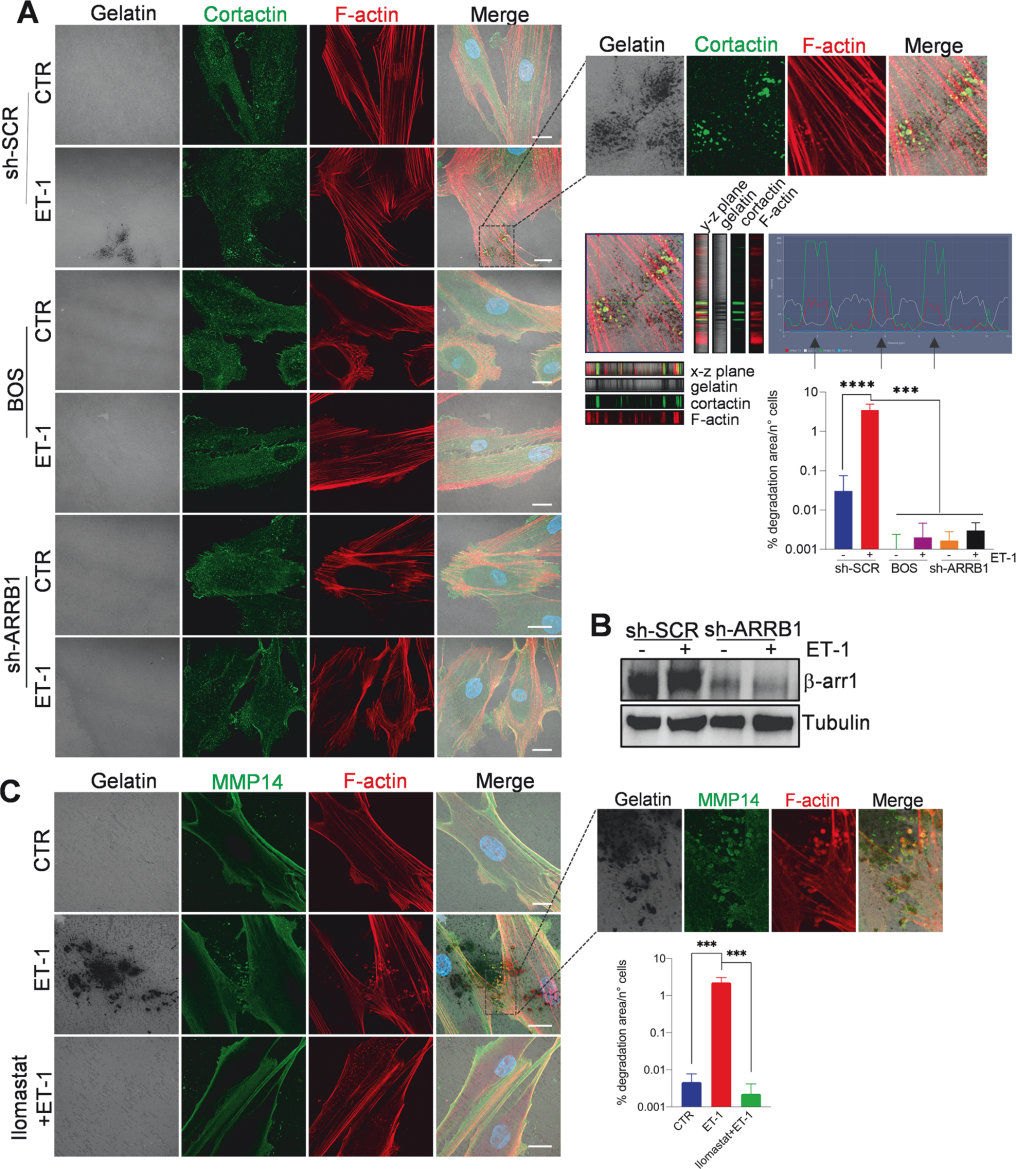
ET-1 regulates invadosome formation and activation. **A** Confocal laser scanning microscopy **(**CLSM) analysis of sh-SCR or sh-ARRB1 HOFs, plated onto gelatin and treated with ET-1 and/or BOS (ET_A/B_R antagonist) for 48 h. Cells were stained for Cortactin (green) and F-actin (red), nuclei in blue (DAPI). Gelatin was reported in pseudo-color gray. Degradation areas appear as black holes, while co-localization is shown in merged images (pseudo-color yellow). Orthogonal views (y-z plane; x-z plane) indicate areas of degraded gelatin with co-localized F-actin and cortactin. Right, separate channels and merged images of the selected ROI and the histogram profiles of F-actin/cortactin/gelatin signals in the line drawn. **B** Representative WB of whole cell lysates from sh-SCR or sh-ARRB1 HOFs stimulated or not with ET-1 and probed with Ab to β-arr1. Tubulin, loading control. **C** CLSM analysis of HOFs, plated onto gelatin and treated with ET-1 or Ilomastat (MMP inhibitor). Cells were stained for MMP14 (green) and F-actin (red). Co-localization is shown in merged images (pseudo-color white or yellow). Right, separate channels and merged images of the selected ROI. Scale bar, 20 μm. Histograms (**A**, **C**) represent the mean ± SD of the normalized degradation area percentage of cells; statistics were obtained using the One-way ANOVA.

Considering the role of invadosome in fibroblast-driven tissue invasion and matrix remodeling [33], and that ET-1/β-arr1 promotes invadopodia in cancer cells [29, 30], we evaluated the ET-1 axis as a regulator of invadosome. As shown by confocal laser scanning microscopy (CLSM) analysis, while untreated HOFs did not form degradative cortactin clusters, under the addition of ET-1, F-actin/cortactin puncta became visible within the cell body, displaying invadosome properties, as degradative activity, but not when cells were treated with AMB or BQ788, or BOS (Fig. 2A and Supplementary Fig. 2). Moreover, β-arr1 silencing inhibits invadosome activity at the same extent of ET-1 receptor antagonists (Fig. 2A, B and Supplementary Fig. 3A, B), while these effects are rescued by overexpression of β-arr1-FLAG (Supplementary Fig. 3A, B), confirming the role of β-arr1 in ET-1-dependent invadosome. The membrane-bound metalloproteinase MT1-MMP (MMP14) regulates invadosome by enabling the degradation of ECM [33]. ET-1 promotes enhanced expression of MMP14 in HOFs (Supplementary Fig. 4A). In invadosome, localization of MMP14 is evident at the substrate-digested dots in ET-1-treated cells, while Ilomastat, an MMP inhibitor, significantly inhibits ET-1-induced gelatin degradation (Fig. 2C), demonstrating the involvement of MMPs in this effect.

**Fig. 3:**
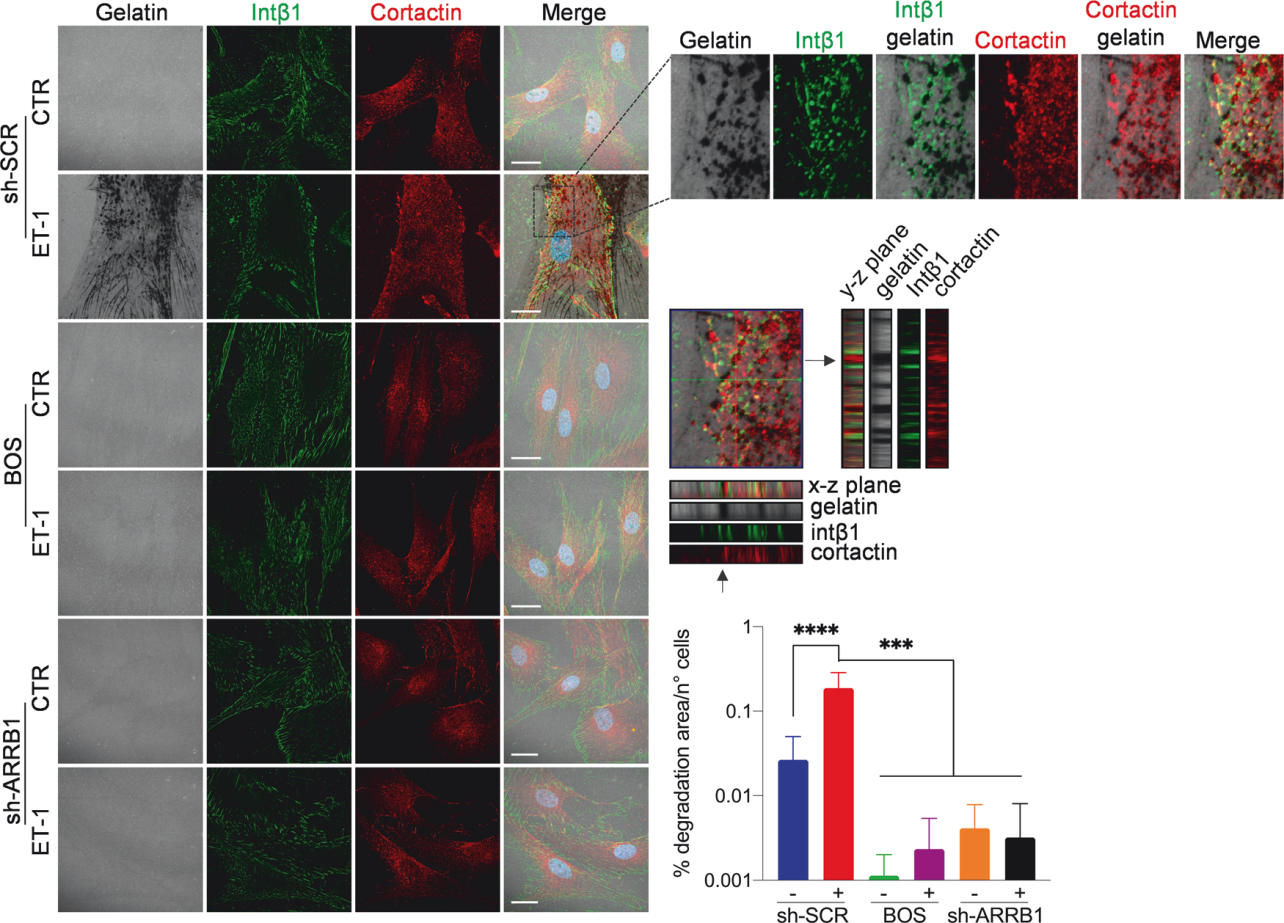
ET-1/ET_A/B_R/β-arr1 axis regulates invadosome activity through Intβ1. CLSM analysis of sh-SCR and sh-ARRB1 HOFs, plated onto gelatin and treated with ET-1 and/or BOS. Cells were stained for Intβ1 (green) and Cortactin (red), nuclei in blue (DAPI). Gelatin was reported in pseudo-color gray. Degradation areas appear as black holes, while co-localization is shown in merged images. Orthogonal views (y-z plane; x-z plane) indicate areas of degraded gelatin with cortactin surrounded by Intβ1 rings. *Right*, separate channels and merged images of the selected ROI. Scale bar, 20 μm. Histograms represent the mean ± SD of the normalized degradation area percentage of cells; statistics were obtained using one-way ANOVA.

**Fig. 4:**
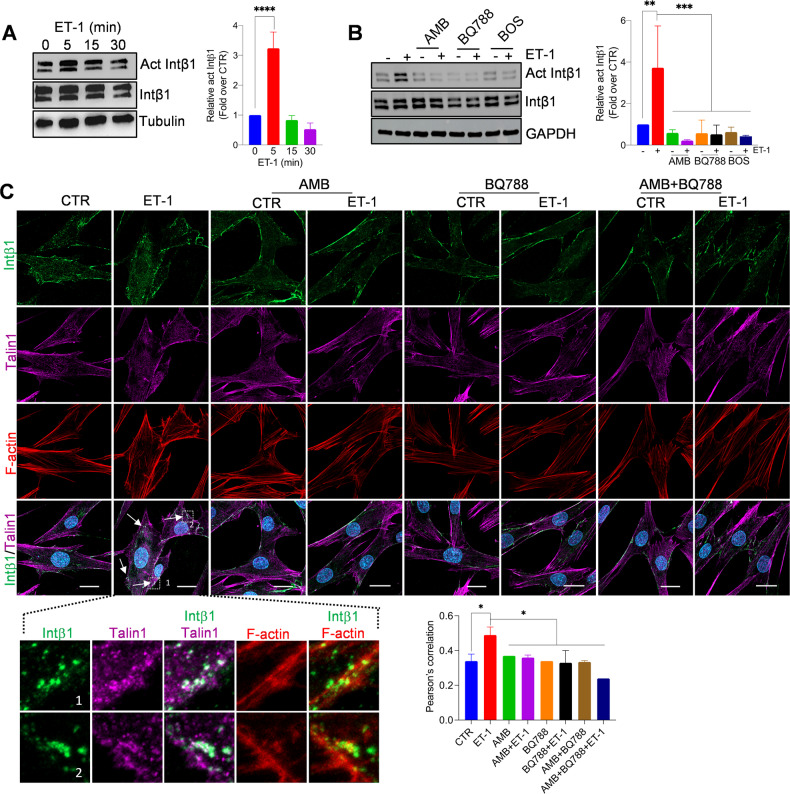
ET-1/ET_A/B_R regulates Intβ1/talin1 signaling through β-arr1. Representative WB of whole cell lysates from HOFs stimulated with **A** ET-1 at indicated times or **B** ET-1 (5 min) and/or AMB and/or BQ788 and/or BOS. Tubulin and GAPDH, loading control. Histograms represent the mean ± SD of densitometric analyses of proteins relative to GAPDH; statistics were obtained using One-way ANOVA. **C** CLSM analysis of HOFs stimulated with ET-1 for 5 min and/or AMB and/or BQ788 and/or AMB + BQ788, stained for active Intβ1 (green), talin1 (magenta) and F-actin (red) detection. Co-localization is shown in merged images (pseudo-color white or yellow). Columns represent the mean ± SD of quantification of Pearson’s correlation between act Intβ1 and talin1; statistics were obtained using one-way ANOVA.

Since F-actin cores are surrounded by “adhesive rings” enriched in integrins, and that β-arr1 regulates both adhesion ring formation around invadopodia, and intβ1 signaling [29, 30, 34–36], we evaluated intβ1-linked molecular effectors to ECM remodeling. HOFs express intβ1 and intα5 (Supplementary Fig. 4B). Intβ1 surrounds cortactin-rich degraded dots in ET-1-treated HOFs, but not in cells treated with AMB, BQ788, BOS, or β-arr1 silencing, to the same extent as ATN161, an intα5β1 antagonist (Fig. 3 and Supplementary Fig. 4C), suggesting the involvement of ET-1-dependent intβ1 signaling in invadosome. Moreover, ET-1 promotes intβ1 activation and downstream related FAK and paxillin (Fig. 4A–C and Supplementary Fig. 4D, 5A, B). This effect is inhibited by the ET-1 receptor antagonists or ATN161 (Fig. 4B and Supplementary Fig. 4D), demonstrating that ET-1 regulates intβ1 signaling. Moreover, β-arr1 silencing inhibits intβ1 activation, and downstream FAK, paxillin, and talin1 phosphorylation (Supplementary Fig. 5A, B), suggesting that ET-1/β-arr1 might contribute to intβ1/talin1 activation. In addition, CLSM analysis shows the recruitment of talin1 to F-actin in ET-1-stimulated cells, but not in cells treated with AMB, BQ788, or in combination (Fig. 4C).

### ET-1-activated CAFs form heterotypic spheroids with high invasive and metastatic behavior

**Fig. 5:**
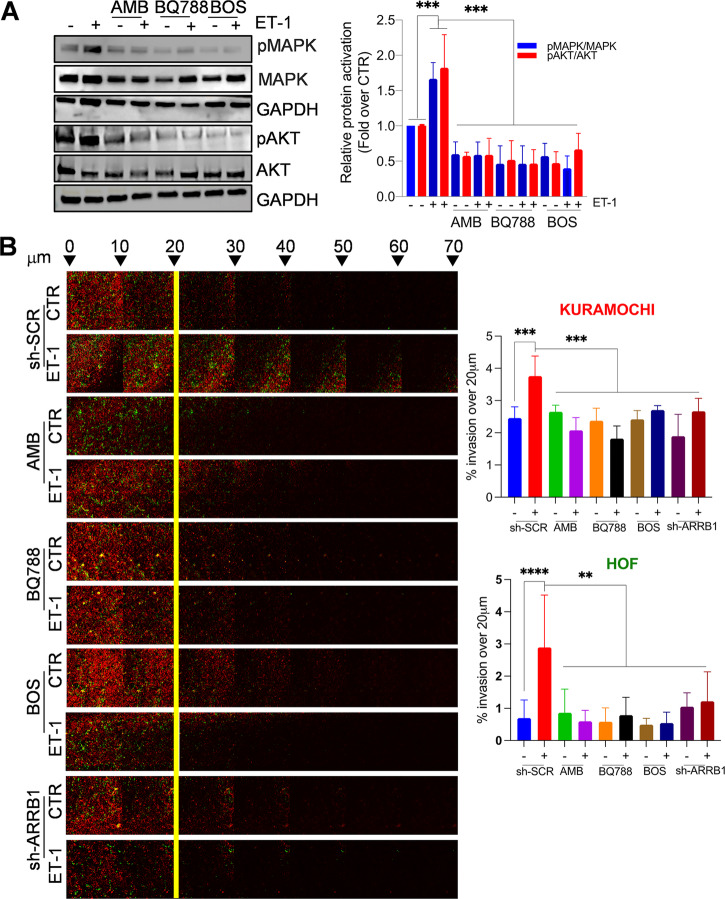
ET-1 facilitates heterotypic spheroid formation, survival, and invasive behavior. **A** Representative WB of whole cell lysates from HOFs, stimulated with ET-1 and/or AMB and/or BQ788 and/or BOS for 5 min, and probed with Abs to pMAPK, MAPK, pAKT and AKT. Histograms represent the mean ± SD of densitometric analyses of proteins relative to GAPDH; statistics were obtained using One-way ANOVA. **B** sh-SCR or sh-ARRB1 Kuramochi/HOFs treated with ET-1 and/or AMB and/or BQ788 and/or BOS were allowed to invade fibronectin/type I collagen plugs in an inverted invasion assay (48 h). Cells were stained with PKH67 (green, HOFs) or PKH26 (red, Kuramochi), before invasion assay, and serial optical sections (10 μm intervals) were acquired. The invasion was measured by dividing the sum of the signal intensity of all slides beyond 20 μm (invading cells) by the sum of the intensity of all slides (total cells); statistics were obtained using One-way ANOVA, Dunnett post hoc analysis. Scale bar, 100 µm.

Then, we evaluated whether ET-1-driven molecular changes translate into HOF phenotypic differences. The addition of ET-1 promotes HOF invasive potential, but not when cells are pretreated with BQ123, BQ788, or BOS, or after β-arr1 silencing (Supplementary Fig. 6A), indicating the involvement of β-arr1 in this process. In addition, a significant release of proteases, such as MMP-7 and uPAR, is observed in ET-1-treated cells (Supplementary Fig. 6B, C). According to the role of ET-1 as a mitogenic factor for cancer and stromal cells [22, 23, 28, 31], cell proliferation and live–dead assays show that ET-1 increases cell vitality, while the addition of AMB, or BQ788 or BOS, as well as silencing of β-arr1 in both cell types, significantly block the basal or ET-1 effects (Supplementary Fig. 6D, E), confirming the presence of an ET-1/ET_A/B_R autocrine loop and the involvement of β-arr1 in cancer-associated ovarian stromal cells. Moreover, a time-dependent activation of p42/44 MAPK and AKT occurs in response to ET-1 stimulation, compared to control cells (Supplementary Fig. 6F). The presence of either ET_A_R or ET_B_R antagonists, or BOS, significantly reduces ET-1-dependent p42/44 MAPK and AKT phosphorylation levels (Fig. 5A), indicating the involvement of both receptors in ET-1-induced mitogenic pathways.

**Fig. 6:**
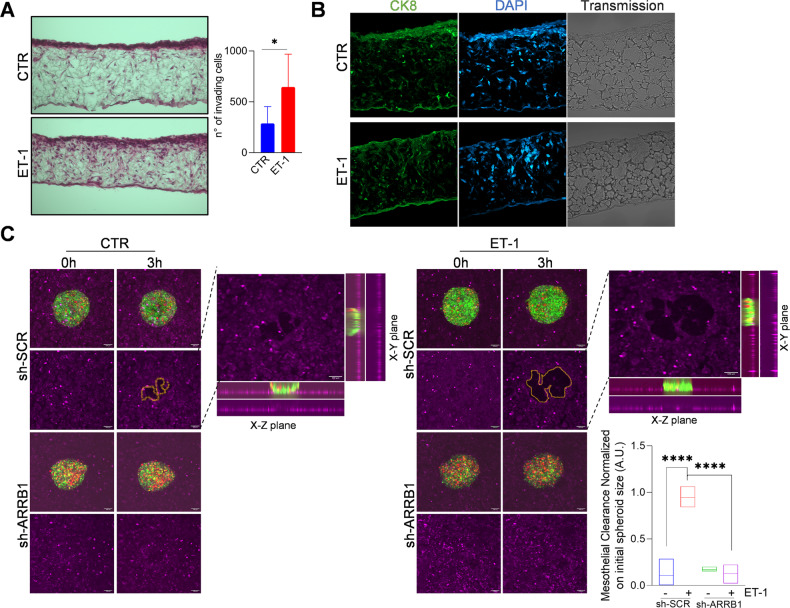
ET-1/β-arr1 enhances the invasive behavior of CAFs/SOC cells. **A** HOFs (6 × 10^5^) and OVCAR3 cells (1.2 × 10^6^) were seeded on a Col1-containing polystyrene scaffold in the absence or presence of ET-1 for 1 week. The images show the invading H&E stained cells after a week in the organotypic model. Columns represent the mean ± SD of the number of invading cells; statistics were obtained using Student *t*-test. **B** Sections as in **A** were stained for CK8 (green) and DAPI (blue) detection. The corresponding transmitted light images are also shown. Arrows depict the top side of the scaffold where cells were plated. **C** Images depict mesothelial clearance induced by sh-SCR-OVCA433 (green)/HOFs (red) or sh-ARRB1-OVCA433 (green)/HOF (red) spheroids treated or not with ET-1 at 0- and 3-h time points. Scale bar, 100 μm. Histograms represent the ratio between the area of the “hole”/aperture in the mesothelial monolayer after 3 h (highlighted with the yellow line) and the initial spheroid area (0 h). One-way ANOVA.

Considering that CAFs serve as a scaffolding for forming heterotypic spheroids [14, 37], we evaluated the potential of the ET-1 axis to induce spheroid formation, supporting mesothelial clearance properties and metastatic behavior. To assess the requirement of ET-1-dependent CAFs to support the ability of cancer cells to invade, we performed a 3D matrix invasion assay in which Kuramochi cells/HOFs were seeded on top of a 100 μm thick containing fibronectin/type I collagen gels. Cells adhered to and invaded these gels, and the addition of ET-1 significantly enhances the average invasion depth into the ECM of both cancer cells and HOFs, whereas this effect is inhibited when cells were treated with AMB, BQ788, BOS, and upon silencing of β-arr1 (Fig. 5B and Supplementary Fig. 7A). We evaluated ET-1-dependent CAF activities in a fibroblast-containing 3D organotypic model composed of a mixture of collagen I and HOFs, and OVCAR3 cells plated on top, by using a polystyrene scaffold engineered into a 200-μm thick membrane. Cells were able to attach, grow, and colonize the full thickness of the scaffold within 7 days of the addition of ET-1, but not in control conditions (Fig. 6A). Immunocytochemical analysis confirms the presence of CK8-expressing SOC cells invading the matrix in ET-1-treated condition (Fig. 6B). Taken together, these findings support the idea that the ET-1 enhances the invasive potential of SOC cells in 3D models containing active fibroblasts.

Then, we evaluated the effect of the ET-1 axis on the ability of heterotypic spheroids to displace mesothelial monolayer. We used sh-SCR OVCA433/HOF or sh-ARRB1 OVCA433/HOF spheroids and fluorescently labeled MCs (Cy5) and followed the dynamics of a mesothelial monolayer after cancer spheroid attachment by live imaging. The cleared area increases when ET-1 was added to the spheroids, but this effect is inhibited after silencing of β-arr1 or in the presence of AMB, BQ788, or BOS (Fig. 6C and Supplementary Fig. 7B). Altogether these data demonstrate that the ET-1/β-arr1 axis contributes to the CAF-dependent invasive and metastatic potential of SOC cells.

### ET_A/B_R blockade inhibits the metastatic potential of combined SOC/HOF cells in vivo

**Fig. 7:**
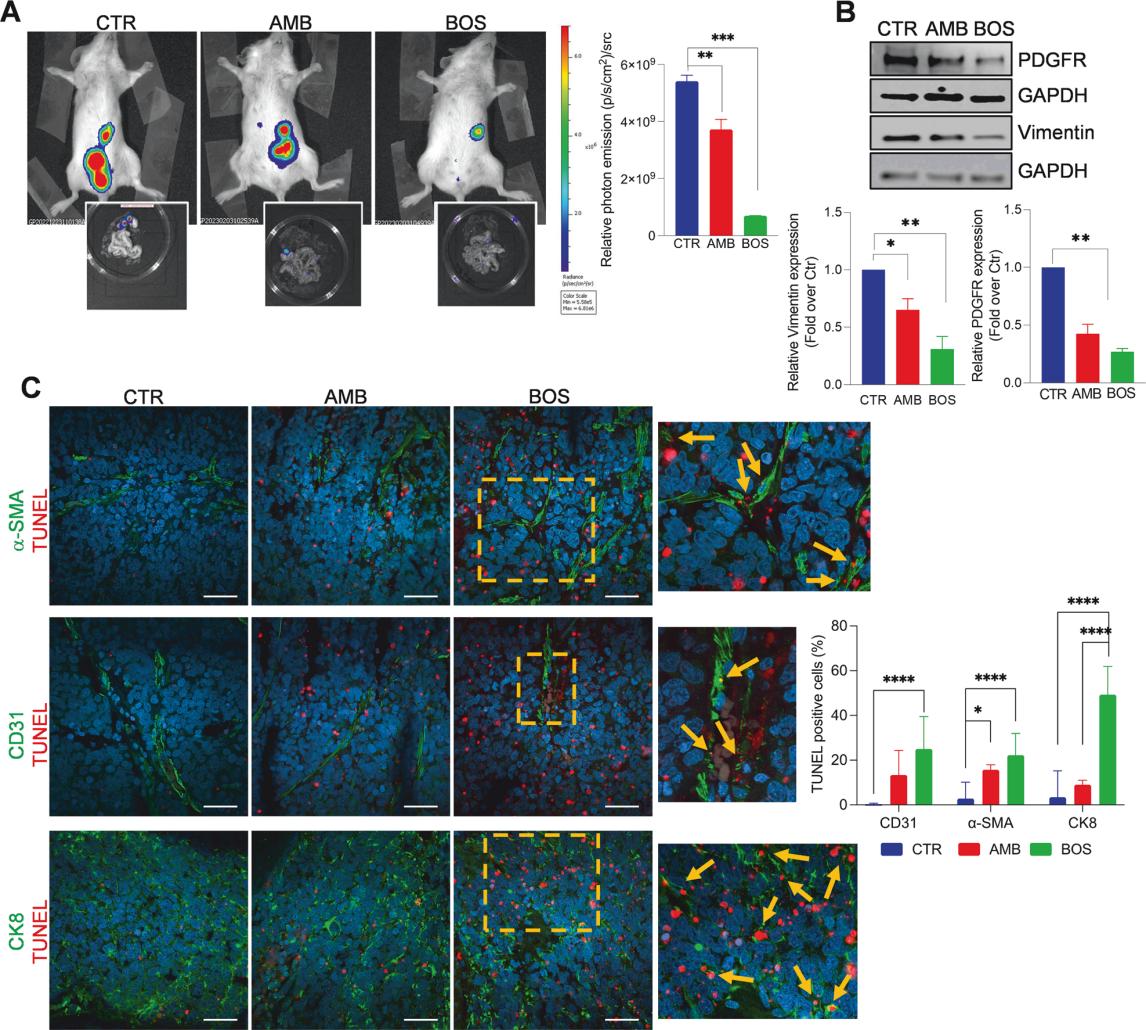
ET_A/B_R blockade suppresses the metastatic potential of CAFs/SOC cells. **A** Bioluminescent images of intraperitoneally (i.p) injected SKOV3-Luc (3 × 10^6^)/HOF-Luc (1 × 10^6^)-cells in NOD/SCID mice, undergoing treatments for 5 weeks with Methocel (vehicle, CTR) or AMB (10 mg/kg, oral daily), BOS (10 mg/kg, i.p. daily). Data are presented as mean ± SD, *n* = 2, one-way ANOVA. Inset, images of i.p. organs visualized by BLI. **B** Representative WB of whole cell lysates from metastatic nodules probed with Abs to PDGFR and Vimentin. Histograms represent the mean ± SD of densitometric analyses of proteins relative to GAPDH; statistics were obtained using one-way ANOVA. **C** CLSM examinations (three-dimensional reconstruction images) of Formalin-Fixed Paraffin-Embedded (FFPE) tissue sections (5 µm thick) from untreated (CTR) and AMB or BOS-treated mice. Sections were stained for TUNEL detection (red) in combination with anti-αSMA (upper panels), anti-PECAM1/CD31 (middle panels) or anti-cytokeratin-8 (lower panels) primary Abs, showed in green. Nuclei were stained with DAPI (blue). Merged images are reported and colocalizations are shown in yellow. Insets represent higher-power magnification images of selected areas with TUNEL staining in proximity of the specific markers (arrows), indicating apoptotic events in tissues from BOS-treated mice. Scale bar, 50 µm. *n* = 2. Histograms represent the ratio of the TUNEL-positive cells in the region of interest to the TUNEL-positive cells in the whole section relative to the number of cells (nuclei). One-way ANOVA.

We, therefore, investigated the involvement of ET-1-driven CAFs in mediating early transcoelomic metastasis and tested the possibility that therapeutic interventions targeting ET-1 receptors could disrupt the interplay between cancer cells and CAFs, preventing metastatic implantation in the peritoneum and beyond. Luc-transfected SKOV3/HOFs were implanted intraperitoneally in mice, then treated with AMB or BOS or control for 5 weeks. Tumor growth in the peritoneal cavity was assessed using BLI images every seven days. Results show that while AMB inhibited intraperitoneal spreading, this effect was significantly pronounced after treatment with BOS (Fig. 7A). Peritoneal metastatic organs were examined, and nodules were used for WB analysis. Reduced expression of PDGFR, and Vimentin is visible in AMB-treated tissues and to a greater extent in those from BOS-treated mice (Fig. 7B). To evaluate whether the effect of antagonists on metastasis regression is related to increased apoptosis, we performed TUNEL assays on tumor sections immunostained for α-SMA, a fibroblast marker, CD31, a marker of endothelial cells, and CK8 for cancer cells. As shown in Fig. 7C, both antagonists significantly enhance the number of apoptotic cells, with a more efficacy effect of BOS on cancer and stromal cells.

The findings indicate a potential treatment window for SOC metastasis by blocking ET_A/B_R, inhibiting crosstalk between cancer cells and CAFs, and enhancing cell death.

## Discussion

In recent years, the TME has been viewed not only as a physical support for cancer cells but also as a facilitator of tumor progression, where changes in stromal cell signaling may precede or act independently in cancer cells to determine disease outcomes [38]. One of the first cell types identified is the resident fibroblast, which can be recruited and activated by the crosstalk with cancer cells. Fibroblasts can establish a niche where they can thrive unrestricted, modifying the physical, dimensional, and chemical aspects of the tissue in a continuous dynamic and co-evolutionary manner with cancer cells. This highlights the need to understand in depth how resident fibroblasts become CAFs and how their functions are altered to establish a mature TME during tumor evolution.

This study provides evidence that the ET_A/B_R/β-arr1 pathway acts as a novel cue for HOFs to become CAFs. This is the result of 1. increased proliferation, migration, and secretion of specific soluble factors. 2. the ability to locally degrade and invade the ECM by facilitating invadosome formation. 3. the formation of heterotypic spheroids with SOC cells with high metastatic potential. At the translational level, ET_A/B_R blockade inhibits tumor invasion by reducing CAF activation and the invasive and metastatic behavior of heterotypic spheroids within a tumor, with reduced cell survival.

The abundant stromal response that accompanies human tumor progression has led to the study of the intriguing properties of fibroblasts, revealing their ability to sense external cues and respond rapidly by activating signaling cascades and switching to an activated state [38, 39]. Several soluble factors derived from the tumor or stromal cells can reinforce a self-sustaining cycle leading to support the plasticity that accompanies the establishment of the CAF phenotype. Previous studies have identified the direct role of the ET-1 axis in driving molecular changes in cancer-associated fibroblasts. In colorectal cancer, ET-1/ET_A_R stimulates proliferative, migratory, and contractile tumorigenic effects [40]. Moreover, specific antagonism of ET_A_R, ET_B_R, or both, inhibits the proliferation, migration, and collagen contraction [28]. In an orthotopic model of breast cancer, the nanoparticles bearing the ET_A/B_R antagonist macitentan prevent fibrotic progression by regulating the CAF function [27]. Notably, ET-1-dependent fibrocyte-associated mechanisms promote lung tumor growth and metastasis, with local upregulation of ET-1 axis members, and bosentan interferes with all phenotypic switches that characterize the lung cancer-supportive niche [26]. In HG-SOC cells, ET-1 dictates the activation of a p53/YAP/HIF-1α transcriptional apparatus in fibroblasts, enhancing the release of VEGF circuits, regulating the DNA damage response, and the efficacy of PARPi treatment [32]. Our study extends these findings in HOFs, by confirming the role of ET-1 as a soluble factor produced by fibroblasts or tumor cells, with both ET_A_R and ET_B_R as part of a feedback loop to enhance their activation. In addition, we provide a previously unrecognized role of β-arr1 in their activation and a new dimension in ECM remodeling via invadosome. Despite the recognized role of β-arr1 in stromal cells in pathological conditions, few studies have demonstrated its involvement in cancer-associated stromal cells. Breast cancer cells promote the migration of fibroblasts, which is associated with increased expression of *β-*arr1 and a small-molecule ligand of *β*-arr1 interferes with the *β*-arr1-cofilin scaffolding pathway, thus inhibiting cancer-dependent migration of fibroblasts [31]. Regarding the SOC, the ET-1/β-arr1 axis triggers the genetic reprogramming of MCs, allowing them to acquire a fibroblast-like phenotype that promotes cancer progression [24]. Here, we report that an ET-1/β-arr1 loop facilitates a proliferative and activated phenotype of fibroblasts via both ET_A/B_R, conferring a pro-tumorigenic role via matrix degradation. The central role of CAF in the TME determined an effort to understand the origins and functions of heterogeneous and distinct populations of CAFs, although there is a lack of consensus markers for specific cell populations [41, 42]. Recent studies in this tumor identified subtypes associated with worse survival, showing mesenchymal phenotype, or associated with stromal response and extensive desmoplasia, acting as contributors to ECM secretion and matrisome expression [42, 43]. Although it is difficult to identify specific subtypes of CAFs induced by ET-1 without omics studies, we hypothesize that ET-1 induces the reprogramming of HOFs to ECM-remodeling/myofibroblastic CAFs, since the observed upregulation of ECM proteins, such as Fibronectin, α-SMA, Vimentin, CD29, and proteases. However, data from a cytokine array also indicate a possible induction of an inflammatory phenotype with tumor-promoting functions. A more in-depth study will be performed in this respect. Several studies have shown that fibroblasts form invadosome that regulate mechanosensing and directional migration [17–21]. The invadosome allows cells to sense and interact with their TME, and participate in its remodeling, although cells of mesenchymal origin, including CAFs, could degrade the surrounding matrix by a mechanism independent of invadosome [44–47]. In addition, some growth factors may induce invadosome in fibrosis-related disease, but few studies have demonstrated factors involved in regulating CAF invadosome. In pancreatic cancer, palladin induces invadosome formation in CAF and invasion through Cdc42 activity [21].

We report for the first time that the ET-1 axis promotes invadosome in fibroblasts for the localized release of lytic enzymes that participate in the generation of tracks in the ECM. These findings, together with those in cancer cells, support the idea that β-arr1, acting as a subcellular signpost of the ET-1 axis, facilitates the coordinated ability of CAFs and cancer cells to degrade the ECM material and promotes cancer invasion. Furthermore, in line with the idea that activated fibroblasts display cancer cell properties in multiple ways, we show that heterotypic spheroids containing ET-1-driven CAFs exhibit enhanced mesothelial clearance capabilities compared to those in which the ET-1 axis is impaired, confirming their supportive role in SOC dissemination [37]. We took advantage of the 3D models to compare the therapeutic anti-metastatic potential of different ET-1 receptor antagonists as well as β-arr1 silencing in vitro and in vivo. Although previous data have shown that ambrisentan prevents peritoneal metastasis in SOC xenografts [30], our study is the first to demonstrate that bosentan is effective in controlling tumor dissemination by targeting the metastatic potential of heterotypic spheroids, associated with enhanced apoptosis in cancer cells, fibroblasts and endothelial cells expressing ET_B_R, placing them as the cornerstone of peritoneal metastasis. These data support previously published findings demonstrating that the dual ET-1R receptor antagonist macitentan interrupts ET-1-driven pro-survival signals, affecting cancer cells and the feed-forward loops in the TME [32]. As different subsets of CAFs are emerging, being tumor-restraining and tumor-promoting roles, depending on the stage of the tumor and the complex context of the surrounding TME, and distinct subsets of CAFs are interconvertible via manipulation of specific signaling [48], further research is required to understand the full CAF pool in a cancer-dependent manner and precise understanding of the mechanisms governing CAF heterogeneity and plasticity is a prerequisite for therapeutic interventions that selectively target tumor-supporting CAFs. Although additional molecular studies are required to provide detailed evidence of the role of the ET_A/B_R/β-arr1 axis to translate these results on CAFs into clinical practice and ultimately to achieve clinical benefit, our findings establish the pivotal role of this signaling pathway in the malignant properties of ovarian fibroblasts, including their ability to degrade ECM via invadosome formation. Considering that CAFs are an ideal target for direct antineoplastic therapies in SOC, our data suggest that ET_A/B_R approaches, targeting both cancer cells and stromal CAFs, may be an effective therapeutic strategy to destroy the survival and invasive behavior of metastatic units, limiting SOC progression.

## Materials and methods

### Cells

HOFs were obtained from ScienCell (Cat# 7330-SC) and cultured in Fibroblast Medium (Cat# 2301-SC; ScienCell). CAFs were obtained from Vitro Biopharma (Cat# CAF02) and cultured in Low-Serum, VitroPlus III, Complete Medium (Cat# PC00B1). Omental-derived adult primary MCs were obtained from Zen-Bio (USA) and cultured in Mesothelial Cell Growth Medium (Cat# MSO-1; Zen-Bio). The SOC cell line SKOV3 (ATCC® HTB-77™) provided by the American Type Culture Collection (LGC Standards, Teddington, UK), was maintained in McCoy’s 5A medium (Cat# 26600-023; GIBCO Thermo Fisher). OVCA433 cell line was provided by Prof. G. Scambia (Catholic University School of Medicine) and maintained in Dulbecco’s modified Eagle medium (Cat# 21885-025; GIBCO Thermo Fisher). Kuramochi cell line was provided by the National Institutes of Biomedical Innovation, Health and Nutrition (NIBIOHN, Osaka, Japan), and the OVCAR3 cells (ATCC® HTB-161 were obtained from the American Type Culture Collection (LGC Standards, Teddington, UK), both cultured in RPMI-1640 medium (Cat# 618700-010; GIBCO Thermo Fisher). All media were supplemented with 10% or 20% FBS, containing penicillin (10,000 U/ml)-streptomycin (10 mg/ml). Cells were incubated at 37 °C in a humidified atmosphere containing 5% CO_2_ and were tested for the absence of viral/mycoplasma contamination.

### Antibodies and chemical reagents

Antibodies (Abs) used for western blotting (WB) were: anti-β-arr1 (Cat# ab32099; Abcam), anti-Tubulin (cat# sc-32293; RRID:AB_628412 ; Santa Cruz), anti-Endothelin A receptor (Cat# PA3-065; Thermo Fisher), anti-Endothelin B receptor (Cat# ab11759; Abcam), anti-GAPDH (Cat# G945; RRID:AB_10597731; Sigma-Aldrich), anti-phospho-paxillin (Cat# ABP-0156; Immunological Sciences), anti-paxillin (cat# MAB-80128; RRID:AB_11187804; Immunological Sciences), anti-phospho-FAK (Cat# ABP-0290; RRID:AB_2173671; Immunological Sciences), anti-FAK (Cat# MAB-10157, RRID:AB_10905163; Immunological Sciences), anti-Intα5 [EPR7854] (Cat# ab150361; Abcam), anti-α-SMA (Cat# NCL-L-SMA; Leica), anti-FAP (Cat# 28244; Abcam), anti-PDGFR (Cat# 32570; Abcam), anti-CD29 (active intβ1) (9EG7) (Cat# 550531; RRID:AB_393729; BD Biosciences), anti-CD29 (intβ1) (Cat#610468; BD Biosciences); anti-pp42/44 MAPK (Cat# 4370; Cell Signaling), anti-p42/44 MAPK (Cat# 4695; Cell Signaling), anti-pAkt (Cat# 2605; Cell Signaling), anti-Akt (Cat# 2602; Cell Signaling), anti-vimentin (Cat# D21H3, Cell Signaling), anti-phospho-talin1 (Ser425) (Cat# TP171; RRID:AB_2840569; ECM Biosciences), anti-talin1 (Cat# MA5-28133; RRID:AB_2204003; Invitrogen), anti-MMP14 (Cat#IM57L, Oncogene), anti-FLAG (F1804 Sigma-Aldrich). Secondary antibodies used for WB were as follows: horseradish peroxidase-conjugated goat anti-rabbit (Cat# 32460, Life Technologies) or anti-mouse (Cat# PA128568, Life Technologies). Primary antibodies used for immunofluorescence (IF) were as follows: anti-Cortactin (Cat# 3503; RRID:AB_2115160; Cell Signaling Technology), anti-CD29 (9EG7), anti-talin1 (Cat# MA5-28133; RRID:AB_2204003; Invitrogen), anti-PECAM1/CD31 (Cat# LS-C286337 Lifespan Biosciences), anti-α-SMA (Cat# NCL-L-SMA; Leica), anti-CK8 (Cat# CK8-TS1-L; Leica), anti-FLAG (F1804 Sigma-Aldrich), anti-MMP14 (Cat#IM57L, Oncogene).

The chemical reagents used were:4’,6’-diamidino-2-phenykindole (DAPI) (Cat# 1331762; Bio-Rad Laboratories), Vectashield (Cat# H-1000; Vector Laboratories), QCM Gelatin Invadopodia Assay (RED) (Cat# ECM671; Millipore), PKH26 red fluorescent cell linker kit for general cell membrane labeling (Cat# PKH26GL-1KT, Sigma-Aldrich), PKH67 green fluorescent cell linker kit for general cell membrane labeling (Cat# PKH67GL-1KT, Sigma- Aldrich), CellTracker™ Deep Red Dye (Cat# C34565,Thermo Fisher), Alexa Fluor-488 phalloidin (Cat # A12379; Thermo Fisher), Alexa Fluor 594 phalloidin (Cat# A12381; Thermo Fisher), Alexa Fluor 633 phalloidin (Cat# A22284; Thermo Fisher), ET-1 (100 nmol/L) (Cat# E7764-1MG; Sigma-Aldrich), BQ788 (1 µM) (Peninsula Laboratories), BQ123 (1 µM) (Cat# L01435, Alexis Corporation), Ambrisentan (1 µM) (Cat# SML2104; Sigma-Aldrich) also called (+) - (2S) - 2-[(4,6dimethylpyrimidin-2-yl) oxy]-3-methoxy-3,3-diphenylpropanoic acid, Bosentan (Cat# HY-A0013A, Med Chem Express), type I collagen rat tail (Cat# MA01730; BD Biosciences), Ilomastat 10 μM (Millipore, Cat# 6M6001), fibronectin human foreskin 15 μg/ml (Cat# F2518-5MG; BD Biosciences), click-iT Plus TUNEL Assay protocol (Invitrogen, Cat# C10247). ET-1R antagonists were added 20 min before the addition of ET-1.

### Cell viability

Cells (3 × 10^4^) were cultured on a 24-well plate and cultured in serum-free medium in different experimental conditions for 48 h. Total cells from each well were collected by using Trypsin-EDTA 1X in PBS (Euroclone) solution and counted by using an automated cell counter (Beckman Coulter). The experiment was performed in sextuplicate for all conditions described and repeated at least three times.

### Live–dead assay

Cells (1 × 10^3^) were cultured in a complete medium. After 24 h of starvation, serum-free medium or serum-free medium containing different experimental treatments were added. After 48 h, the Cyto3DTM Live–Dead Assay Kit (TheWell Bioscience, Inc., North Brunswick, NJ, USA) was used to determine the live/dead nucleated cells using a dual-fluorescence system of acridine orange (AO) and propidium iodide (PI), both nuclear staining (nucleic acid binding) dyes. All live nucleated cells (green), and all dead nucleated cells (red) were counted. Several images were obtained using a Bio-Rad ZOE fluorescent cell imager (Bio-Rad Laboratories). Quantification analyses were performed using ImageJ (https://imagej.net/software/fiji), a Java-based freeware, analyzing the mean gray value of the green and red channels separately, and then calculating the green/red ratio.

### ELISA

Following the manufacturer’s protocols, uPAR, MMP-7, and ET-1 secretion were evaluated by using a Human uPAR Immunoassay (Cat# DUP00 R&D), Human Total MMP-7 Quantikine ELISA Kit (Cat# DMP700, R&D), and ET-1 Human Endothelin-1 ELISA Kit (Car# CEK1146, Cohesion Biosciences), respectively. Briefly, 50 µL of conditioned medium obtained at different time points was added into each well of the 96-well plate pre-coated with anti-tag antibody by using the manufacturer’s instructions. The concentration of each desired protein in each sample was determined by interpolating the absorbance values against the standard curve that was calculated by recombinant proteins at gradient dilution.

### Silencing of β-arr1 and transient transfection

To obtain stable silencing, HOFs, OVCA433, and Kuramochi cells were infected with shRNA-expressing lentiviruses. Lentiviral particles were obtained by co-transfecting HEK293FT cells (from ATCC) with pMD2.G and psPAX2 plasmids (Addgene #12259 and #12260 respectively), and the target lentiviral vectors pLV[Exp]-U6>Scramble[shRNA#1]-hPGK>Puro(ns):T2A:Luciferase or pLV[Exp]-U6 > hARRB1[shRNA#1]-hPGK>Puro(ns):T2A:Luciferase (Vector Builder) with JetPEI transfection reagent (PolyPlus, Graffenstaden, FR) according to the manufacturer’s instructions. After 48 h, the HEK293FT supernatant, containing infecting particles, was collected, and replaced with a fresh medium. The collected supernatant was centrifuged to eliminate cell debris and directly administered to cells seeded at 30–40% confluence. A second infection cycle was repeated after 24 h. The cells were then allowed to recover in fresh medium for 48 h and then splitted. Lentivirus-infected cells were selected for 72 h in medium containing 1ug/ml of puromycin (Cat #A11138-03, Gibco). The pcDNA3-β-arr1-FLAG wild-type plasmid construct, a ‘wobble’ mutant construct encoding rat β-arr1 sequences resistant to small interfering RNA targeting kindly provided by Dr Robert Lefkowitz (Howard Hughes Medical Institute, Duke University, Durham, NC, USA) was for the ectopic expression of β-arr1, or empty vector.

### RNA isolation and qRT-PCR

Total RNA was extracted from cells using PureZol (Cat# 7326880 BioRad), according to the manufacturer’s instructions and 1 µg was used for retrotranscription (RT) using PrimeScrip RT Reagent Kit (Cat# RR037A, Takara). Quantitative real-time PCR was performed by using the light Cycler QuantStudio 3 qPCR System (Applied Biosystem) using SensiFAST™ SYBR® Hi-ROX One-Step Kit (Meridian Bioscience). Final data were obtained by using 2^-ΔΔCt^ method. The number of each gene-amplified product was normalized to the number of GAPDH amplified products. The primers used were as follows:

EDNRA F: 5′-ATCACCGTCCTCAACCTCT-3′

EDNRA R: 5′-CAGTGGAGAGACAATTTCAATGGC-3′

EDNRB F: 5′-ATCACCGTCCTCAACCTCT-3′

EDNRB R: 5′-CAGATGGAGAGACAATTTCAATGGC-3′

ARRB1 F: 5′-CAGGAACGCCTCATCAAGA-3′

ARRB1 R: 5′-GCAGTGTCACAGAACATGGA-3′

EDN1 F: 5′-GTGTCTACTTCTGCCACCTG-3′

EDN1 R: 5′-AAGTAAATTCTCAAGGCTCTCT-3′

GAPDH F: 5′-ACATCGCTCAGACACCATG-3

GAPDH R: 5′-TGTAGTTGAGGTCAATGAAGGGG-3′

### Western blotting (WB)

For WB analysis, total cells were detached by scraping, collected by centrifugation, and lysed in RIPA buffer [50 mMTris·HCl (pH 7.5), 150 mm NaCl, 1% Nonidet P-40, 0.5% sodium deoxycholate (NaDoc), 0.1% SDS] containing proteases and phosphatase inhibitors (Roche). Protein concentrations were determined using the DC Protein assay (Bio-Rad Laboratories). Cell lysates were resolved on MiniPROTEAN TGX gels and transferred to nitrocellulose membranes (Bio-Rad Laboratories), followed by WB using indicated primary Abs and revealed by using horseradish peroxidase-conjugated goat anti-rabbit or anti-mouse Abs (Bio-Rad Laboratories). Proteins were visualized by chemiluminescence (Clarity Western ECL Substrates, Bio-Rad Laboratories) by using Azure 300 (Azure Biosystems) or by ChemiDoc Imaging System and Image Lab Software (Biorad Laboratories). Quantification analyses were performed using ImageJ and reflected the relative amounts as a ratio of each protein band relative to the loading control of the lane.

### Human Cytokine array

A Human Cytokine Array Kit (ARY026) was purchased from R&D (Minneapolis, USA). Briefly, the conditioned medium was added into Array Buffers (R&D, ARY026) to a final volume of 1.5 mL and then applied to each antibody-printed nitrocellulose membrane for overnight incubation at 4 °C. On the next day, the membrane was incubated with the detection antibody cocktail (R&D, ARY026) for 1 h at room temperature. Treated with HRP-conjugated streptavidin antibody, the membrane was exposed to ChemiDoc. Each pair of positive dots represented signals of highly expressed cytokines and the intensity was quantified by ImageJ software. The full list of all proteins candidates is available at the manufacturer’s official website (Proteome Profiler Human XL Oncology Array ARY026: R&D Systems (rndsystems.com). Data extraction and analysis were performed after subtraction of the background and normalization to the internal references provided by the manufacturer, using an ImageJ protein array analyzer software (http://rsb.info.nih.gov/ij/macros/toolsets/Dot%20Blot%20Analyzer.txt).

### Immunofluorescence

Cells cultured on coverslips were fixed with 4% paraformaldehyde for 10 min at room temperature, permeabilized with 0.2% Triton-X-100 and blocked with 0.1 M glycine, 1% BSA and 0.1% Tween20 in PBS for 30 min at room temperature. Samples were incubated with primary Abs in 0,5% BSA in PBS overnight at 4 °C, followed by incubation with secondary Abs conjugated with suitable fluorochromes, as previously described. Nuclei were stained with DAPI. Coverslips were finally mounted with a Vectashield mounting medium for fluorescence. CLSM observations were performed with a Zeiss LSM980 apparatus, using a 63x/1.40 NA oil objective and excitation spectral laser lines at 405, 488, 543, 594, and 639 nm. Image acquisition and processing were carried out using the Zeiss Confocal Software Zen 3.1 (Blue edition). Signals from different fluorescent probes were taken in sequential scan settings and co-localization was visualized in merge images. Several cells were analyzed for each labeling condition, and representative results were shown.

All co-localization analyses were carried out using the Coloc2 plugin of ImageJ software to calculate Pearson’s correlation coefficients. This software estimates the degree of overlap between fluorescence signals obtained in two separate fluorescent channels. The Pearson’s coefficients were calculated from multiple images and then averaged, and SD of the mean was calculated.

### Immunofluorescence on tumor tissue sections

Formalin-fixed paraffin-embedded (FFPE) tissue sections (5 µm thick) were deparaffinized with xylene and ethanol, hydrated through graded alcohols. For TUNEL detection (or staining) in combination with anti-PECAM1/CD31, anti-SMA, or anti*-*Cytokeratin-8 primary Abs, hydrated FFPE tissue sections were first subjected to a heat-induced epitope retrieval step by pH6 citrate buffer (Novus Biologicals) for 3 × 3 min in a microwave oven and then treated according to Click-iT Plus TUNEL Assay protocol (Invitrogen, Cat# C10247). At the end of TUNEL staining sections were blocked in PBS-BSA 3% for 60 min at 37 °C and stained with primary Abs overnight at 4 °C, followed by incubation with Alexa Fluor-488 F(ab)2 fragments of goat anti-mouse or goat anti-rabbit IgG plus DAPI for 60 min at 37 °C (Thermo Fisher Scientific). The slides were mounted in Vectashield (Vector Laboratories) and observed on a Zeiss LSM980 microscope, using a 40x/1.40 NA oil objective and excitation spectral laser lines at 405, 488, and 639 nm. At least 10 photomicrographs were collected from each tissue specimen, two tumors from each group were selected for analysis, and the number of TUNEL-positive staining cells were counted by using FIJI software. From the native RGB digital image, a green channel, a blue channel and an 8-bit red channel were extracted, and background noise was subtracted to obtain a cleaner image. Next, we applied the closest-to-reality (of the original image) thresholding method to obtain a binary image and used “find maxima” tool (noise tolerance = constant for the same set of images, output type = maxima within tolerance) to separately count the number of nuclei, TUNEL-positive cells, and TUNEL-positive cells in the specific ROI around the green-marked cells. The analysis was done by relating the TUNEL-positive cells of interest to the TUNEL-positive cells in the whole section relative to the number of cells (nuclei).

### Transwell invasion assay

The assays were carried out using an insert 8.0 µm pore-sized membranes (Cat# 662638; Greiner Bio-one). Cells (3 × 10^4^) were added to the lower chamber pre-coated with Cultrex Basement Membrane Matrix (Cat# 3500-096-03; Trevigen) and cultured with serum-free medium in different experimental conditions, as indicated. The cells were left to invade for 12 hr at 37 °C. Cells on the upper part of the membrane were scraped using a cotton swab, and the migrated cells were stained using Three-Step Stain Set (Cat# 3300; Thermo Scientific). Each experiment was performed in triplicates for all conditions described and repeated at least three times. From each transwell, several images were taken by using Bio-Rad ZOE fluorescent cell imager under a phase-contrast microscope, and four broad fields were considered for quantification. For each image, foci were quantified using the “Find Maxima” tool in the FIJI software (noise tolerance = constant for the same set of images, output type = maxima within tolerance).

### Fluorescent gelatin degradation assay

Coverslips were inverted on 200 μL drop of QCM Gelatin Invadopodia Assay (Millipore) and heated to 37 °C. Coverslips were fixed in 0.5% glutaraldehyde for 15 min at 4 °C and after washing with PBS. Slides were sterilized with 70% ethanol and left in complete growth media for 1 h before use. Cells were cultured on gelatin-coated coverslips in a 24-well plate and left to adhere. Cells were then incubated for 48 hr in different experimental conditions and finally fixed and stained for CLSM examinations. Fluorescence signals were analyzed by Zeiss LSM980 Microscope equipped with a 63× oil objective. 3D reconstruction images of selected regions of interest (ROI) with evident matrix degradation spots were shown. To quantify invadopodia activity, black-and-white images of gelatin degradation are analyzed using FIJI software. The fraction of degraded area was analyzed in this way: Analyze>Set Measurements>select “Area Fraction”. The “area fraction” value was normalized to the number of nuclei in each image as measured from the DAPI channel in the same field [49].

### Collagen contraction assay

Collagen gel was prepared according to the manufacturer’s protocol. Briefly, collagen solution was neutralized by adding 12 µl of acetic acid 0,1% and 7 µl of 1 M NaOH to 600 µl of collagen type I stock solution (3 mg/mL). Then, 500 µl of cell culture medium containing HOFs (3.5 × 10^3^) were added and gently mixed to reach uniform distribution. The prepared solution was poured into a non-tissue culture-treated 24-well plate and incubated at 37 °C for 30 min. Cells (3.5 × 10^3^) were added to collagen. After collagen polymerization, gels were gently detached from the edges of the culture wells on the sides of the wells using a sterile tip and 1 mL of starvation medium was added to each gel in different experimental conditions. Gels were maintained at 37 °C and 5% CO_2_ in an Okolab cage incubator and monitored by time-lapse microscopy using an epifluorescence inverted microscope (Nikon Eclipse Ti-E). Collagen contraction was measured by NIS Elements AR software (Nikon) and expressed as a percentage of the initial gel area. The collected data were analyzed using GraphPad Prism software and statistical analysis was performed on at least three independent experiments.

### Mesothelial clearance

Sh-SCR OVCA433 cells and HOFs or sh-ARRB1 OVCA433 and HOFs were labeled, respectively, with 2 × 10^6^ M PKH67 green fluorescent cell linker or 2 × 10^6^ M PKH26 red fluorescent cell linker. Spheroids were formed by incubating 1 × 10^3^ cells (3:1, OVCA433:HOFs) per well in a 96-well U-bottom-shaped culture dish with a cell-repelling surface (Cat# F202003, faCellitate) at 37 °C for 16 h. MCs were labeled with Cy5 cell linker and MC monolayer was prepared by plating 40 × 10^3^ cells per well in fibronectin-coated (50 μg/ml) Ibidi chamber slides (μ-slide 96-well Uncoated, Ibidi, Cat# 81811) followed by incubation at 37 °C for 16 hr. The spheroids were then transferred to the slides with the MC monolayers and the three cell populations were imaged. For live imaging experiments, we used an X-Light V3 confocal spinning disk unit (CrestOptics) mounted on a Nikon Ti-E Inverted Motorized time-lapse microscope with an integrated Perfect Focus System and equipped with a Kinetix CMOS camera (Photometrics) and a Celesta laser source (Lumencor). Ibidi slides were placed in the time-lapse microscope incubation chamber with integrated temperature, CO_2,_ and humidity control (OkoLab), and time-lapse Z-stack acquisitions were conducted for 3 h at 30 min intervals using NIS Elements AR ver.5 software (Nikon). Images represent the Maximum Intensity Projection (MIP) of 200 µm Z-stacks (z. step 20 µm). A ROI was selected for each image. For each time point, the non-fluorescent area in the mesothelial monolayer underneath the spheroid was measured using FIJI software and normalized to the initial spheroid area. Experiments were conducted at least in triplicate.

### Inverted 3D collagen invasion assay

One hundred microliters of collagen (2.0 mg/ml type I/fibronectin (15 μg/ml) was allowed to polymerize in transwell inserts (the same as described in “transwell invasion assay”) for 2 h at 37 °C. 3 × 10^4^ Kuramochi cells and 1 × 10^4^ HOFs were stained respectively with PKH26 red fluorescent cell linker and PKH67 green fluorescent cell linker, were seeded on top of the gel in a serum-free medium, and stimuli and/or inhibitors were added to the medium in the bottom chamber of the transwell as chemoattractants. After 48 h, the cells were fixed, stained, and visualized by an X-Light V3 confocal spinning disk unit, as described previously. Images represent 70 µm Z-stacks (z. step 10 µm). The invasion was measured using FIJI software by dividing the sum of the signal intensity of all slides beyond 20 μm (invading cells) by the sum of the intensity of all slides (total cells).

### 3D organotypic cultures

The organotypic model was created by putting type I collagen (0.8 mg/ml) and HOFs (7 × 10^5^) on the Alvetex scaffolds (Reinnervate, Sedgefield, Co. Durham, UK). The day after, OVCAR3 cells (1.3 × 10^6^) were seeded and cultured for 7 days. After 2 days, the growth medium was replaced with a starved medium under the conditions indicated. After 7 days, the scaffolds, once the medium is removed, are washed with PBS, fixed with Bouin’s solution for 16 h, dehydrated with sequential ethanol washes (30–95%), clarified in xylene, and sliced into two parts and embedded on FFPE. The scaffolds were sectioned at 10 μm, deparaffinized with xylene and ethanol, hydrated through graded alcohols, and subjected to heat-induced epitope retrieval step by pH6 citrate buffer (Novus Biologicals) three times for 3 min in a microwave. Sections were washed with PBS-T (0.01% Tween 20) and blocked in PBS-BSA 3% for 30 min at 37 °C, and then stained in two different ways: (1) with the standard hematoxylin (Mayer’s solution for hematoxylin Sigma Aldrich, Cat# MHS16) and eosin (alcoholic Eosin Y solution, Sigma Aldrich, Cat# HT110116l) technique; (2) for Cytokeratin 8 detection (Leica Biosistems, Cat#NCL-L-CK8-TS1) was added to PBS-BSA (0.5% BSA) and incubated 30 min at 37 °C, followed by incubation with Alexa Fluor-488 F(ab)2 fragments of goat anti-rat IgG plus DAPI (Thermo Fisher Scientific). The slides were mounted in Vectashield (Vector Laboratories) and observed on a Zeiss LSM980 confocal laser scanning microscope. Each hematoxylin-eosin image was converted on FIJI to a black-and-white image, and then with Image>Adjust>Threshold, an area with a white background was selected that was representative of the invaded cells in the scaffold. A ROI was selected for each image that included only the cells within the scaffold, i.e., those that invaded, and then they were counted with the “analyze particles” function.

### In vivo experiments

For in vivo animal studies, the experimental protocols complied with the principles of ARRIVE (https://arriveguidelines.org) and were approved by the National Ethics Committee for Animal Experimentation of the Italian Ministry of Health (authorization N1/2020- PR #365869604). The mice were housed in single cages with wood-derived bedding material in a specific pathogen-free facility with a 12 h light/dark cycle under controlled temperatures. The mice were cared for under the principles of laboratory animal care (National, Bethesda, Dof USA no. 85-23, revised 1985) and national laws, and received water and food ad libitum. 4–6 weeks of age female NOD/SCID mice (Charles River Laboratories) were used. Mice were injected intraperitoneally (i.p.) with 200 μL PBS containing 3 × 10^6^ viable of SKOV3-Luc+HOFs-Luc (3:1, SKOV3:HOFs) [30], following the guidelines for animal experimentation. One week after, mice, were randomized into three different groups undergoing the following treatments for 5 weeks: (i) 200 μL Metocell (vehicle oral gavage, CTR), (ii) 200 μL Bosentan (10 mg/kg, oral gavage daily), and Ambrisentan (5 mg/kg, oral gavage daily). Mice were observed 2 times per week and monitored for signs of distress (i.e., changes in appearance, respiration, activity, etc.) and weighed. Mice showing signs of distress or losing greater than 15% of body weight were euthanized. Tumor burden was assessed once per week after tumor cell injection by measuring light emission following intraperitoneal luciferin administration (75 mg/kg body weight, intraperitoneal; Perkin Elmer, Hopkinton, MA, USA). Briefly, 10 min after administration of D-luciferin, photon emission was acquired for 3 min and analyzed with a CCD camera (Xenogen IVIS Lumina System; Perkin Elmer). Total flux (photons/second) was determined for the entire abdominal cavity per mouse and normalized to the mean total flux of control-treated mice imaged one week after tumor cell injection. Upon experimental termination, mice were euthanized, and visible metastases were carefully dissected, frozen, and used for WB, and IF analysis.

### Statistical analysis

All the experiments were repeated at least three times, otherwise indicated. We used cell cultures with a normal distribution and similar variance between groups. Statistical analysis was conducted using GraphPad Prism software and the values represent mean ± SD. Graphs comparing two conditions were analyzed via unpaired t-test with Welch’s correction. Graphs comparing more than two conditions were analyzed via one-way ANOVA followed by Dunnett post hoc analysis. The statistical significance was symbolized by *(*P* ≤ 0.05), **(*P* ≤ 0.01), ***(*P* ≤ 0.001), or ****(*P* ≤ 0.0001).

### Supplementary information

Supplementary figures

Original Data

## Data Availability

Data generated during the current study are included in this article and its supplementary information file and could be available on reasonable request by inquiring the corresponding author. Uncropped western blots can be seen in supplemental materials.
