# Supplementary material for: The β-arrestin1/endothelin axis bolsters ovarian fibroblast-dependent invadosome activity and cancer cell metastatic potential
Source: Cell Death Dis. 2024 May 22;15(5):358. doi: 10.1038/s41419-024-06730-6 (PMC11111729; doi:10.1038/s41419-024-06730-6)
Supplement: Supplementary file 1 — Supplementary figures [file 41419_2024_6730_MOESM1_ESM.pdf]

Supplementary Figures

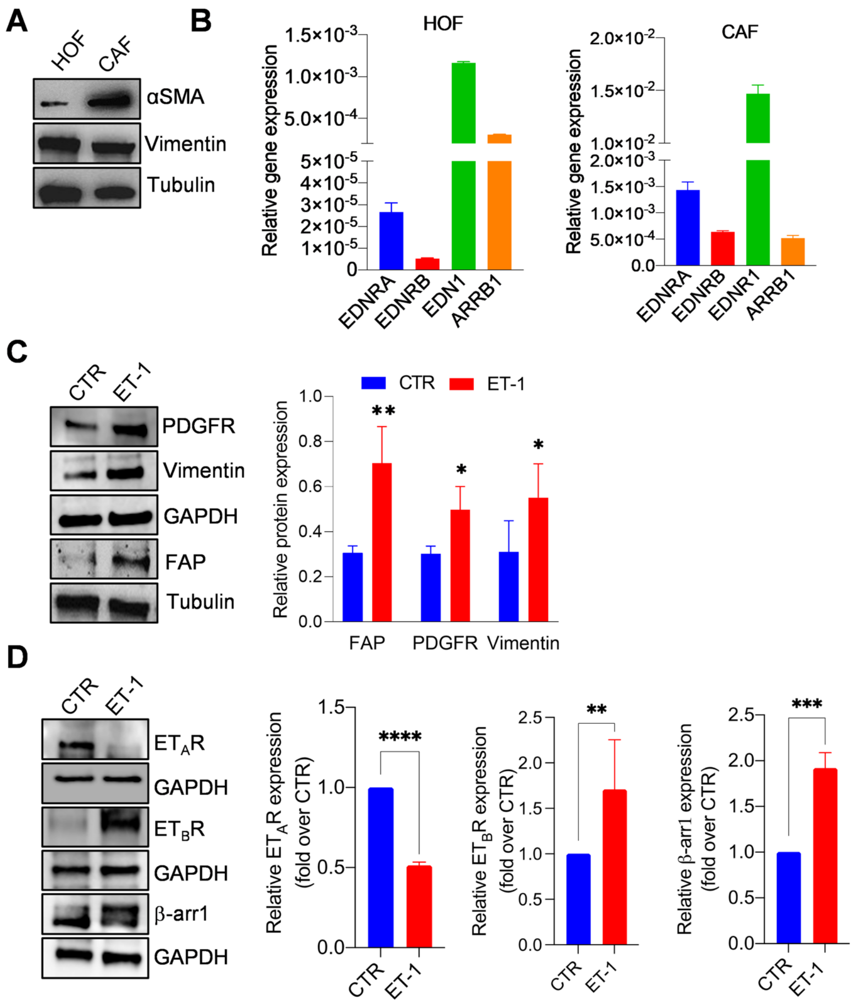

Supplementary Figure 1. ET-1/β-arr1 axis promotes ovarian fibroblast activation. A. Representative Western blotting (WB) of whole cell lysates probed with Abs to αSMA, and Vimentin. Tubulin, loading control. **B.** qPCR analysis of EDN1 (ET-1), EDNRA (ET_AR), EDNRB (ET_BR), and ARRB1 (β-arr1) in HOFs and CAFs. Representative WB of whole cell lysates from HOFs stimulated or not with ET-1 (24 hr) and probed with Abs to **C.** FAP, PDGRF, and Vimentin, or **D.** ET_AR**,** ET_BR and β-arr1. Histograms represent the mean ± SD of densitometric analyses of proteins relative to Tubulin or GAPDH; statistics were obtained using Student t-test.

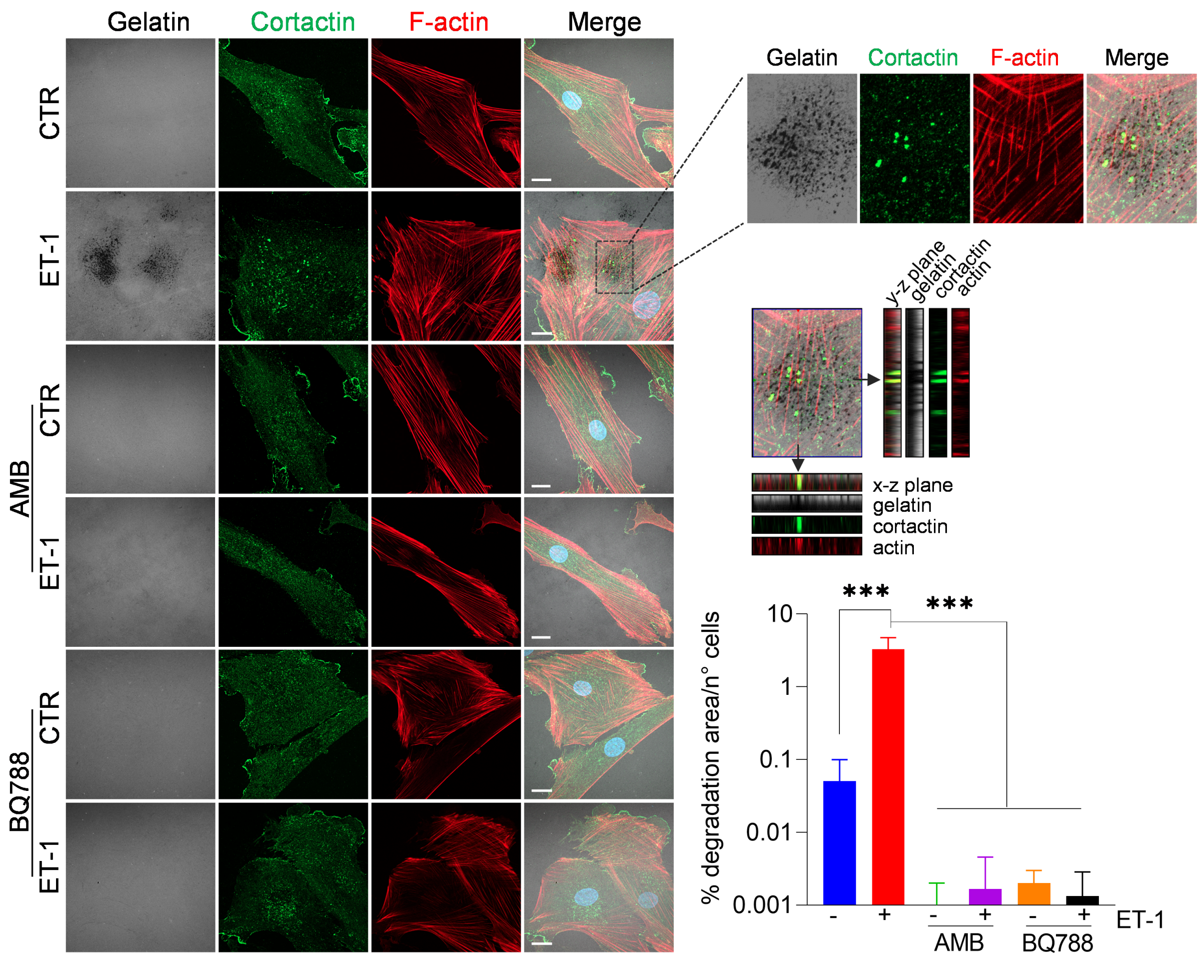

Supplementary Figure 2. ET-1 regulates invadosome formation and activation through $ET_{A/B}R$. Confocal laser microscopy (CLSM) analysis of HOFs, plated onto gelatin and treated with ET-1 and/or AMB and/or BQ788. Cells were stained for Cortactin (green) and F-actin (red), nuclei in blue (DAPI). Gelatin was reported in pseudo color gray. Degradation areas appear as black holes, while co-localization is shown in merged images. Orthogonal views (y-z plane; x-z plane) indicate areas of degraded gelatin with co-localized F-actin and cortactin. *Right*, separate channels and merged images of the selected ROI. Histograms represent the mean ± SD of the normalized degradation area percentage of cells; statistics were obtained using one-way ANOVA.

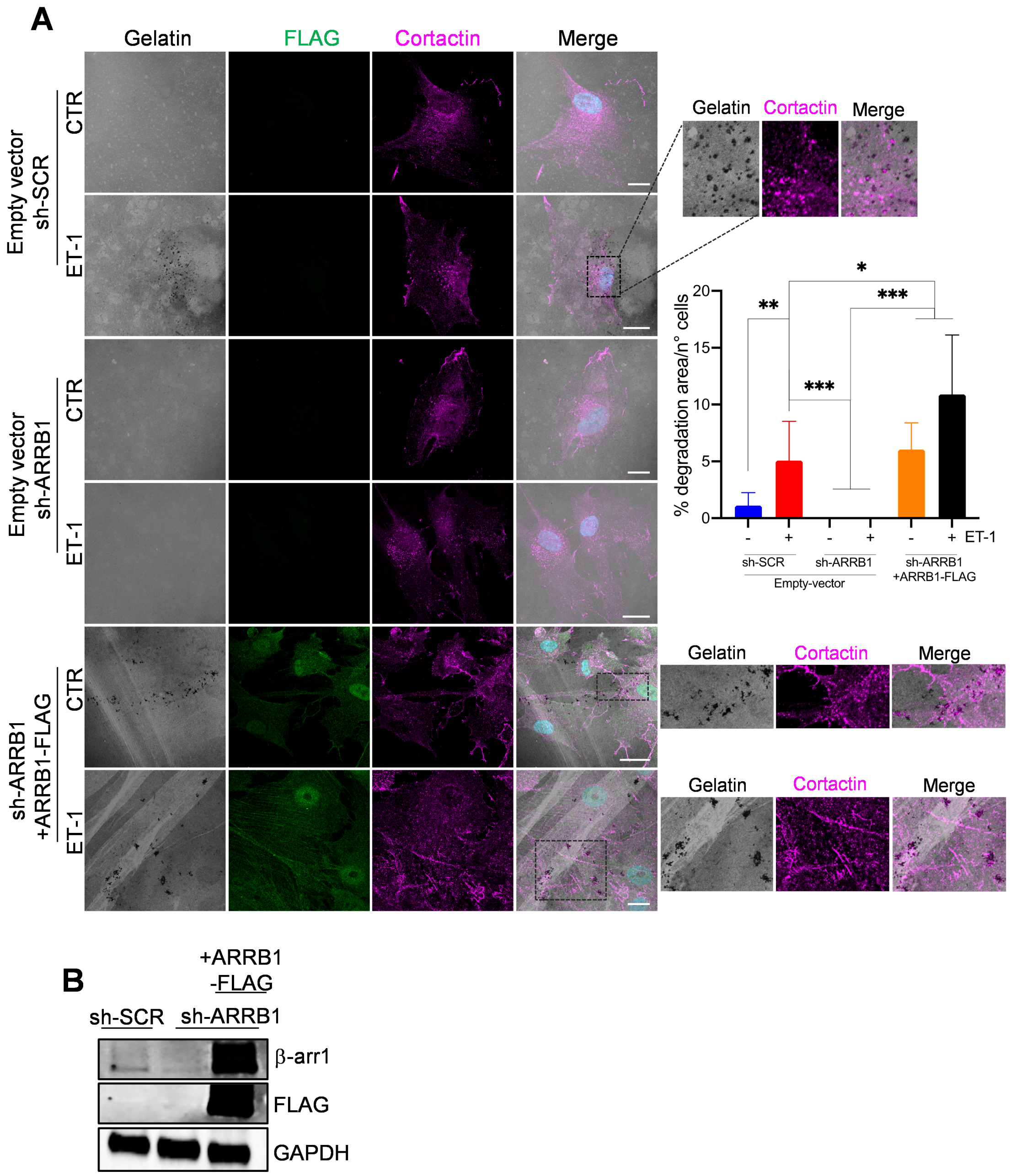

Supplementary Figure 3. **ET-1 regulates invadosome activation through β-arr1. A.** CLSM analysis of sh-SCR and sh-ARRB1-HOFs, or transfected with empty vector or β-arr1-FLAG, plated onto gelatin, and treated with ET-1. Cells were stained for Cortactin (Magenta) and anti-FLAG (green), nuclei in blue (DAPI). Gelatin was reported in pseudo color gray. Degradation areas appear as black holes, while co-localization is shown in merged images. Scale bar, 20 μm. Histograms represent the mean ± SD of the normalized degradation area percentage of cells; statistics were obtained using One-way ANOVA. **B.** Representative WB of whole cell lysates from HOFs transfected as in **A.** probed with Abs to β-arr1 and FLAG. GAPDH, loading control.

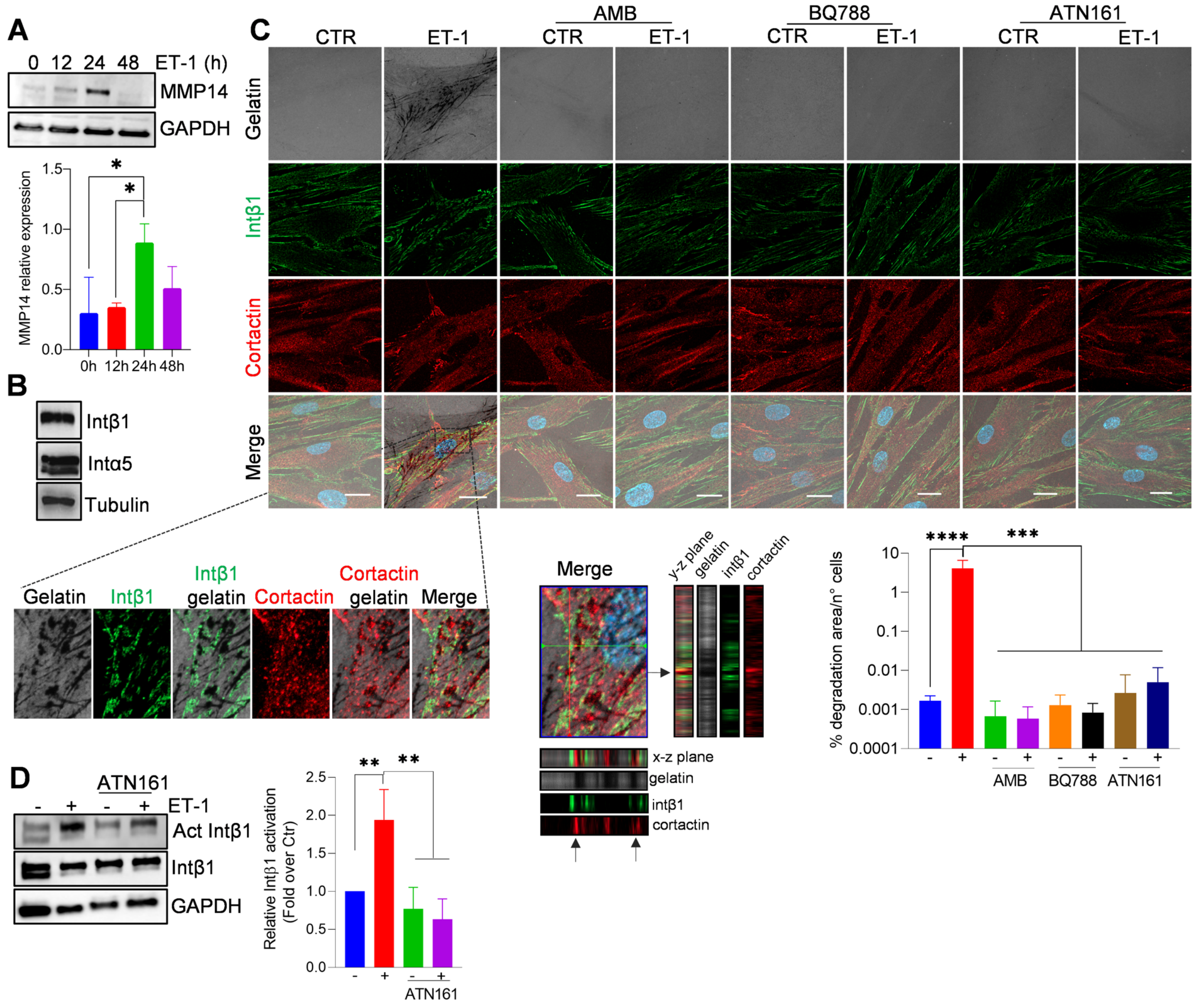

Supplementary Figure 4. ET-1/ET$_{A/B}$R/β-arr1 axis regulates invadosome activity through Intβ1. A. Representative WB of whole cell lysates from HOFs treated with ET-1 at different times, probed with Abs to MMP14. Histograms represent the mean ± SD of densitometric analyses of proteins relative to GAPDH or Tubulin; statistics were obtained using One-way ANOVA. **B.** Representative WB of whole cell lysates from HOFs probed with Abs to intβ1 and intα5. Tubulin, loading control. **C.** CLSM analysis of HOFs, plated onto gelatin and treated with ET-1 and/or AMB and/or BQ788 and/or ATN161. Cells were stained for Intβ1 (green) and Cortactin (red), nuclei in blue (DAPI). Gelatin was reported in pseudo color gray. Degradation areas appear as black holes, while co-localization is shown in merged images. Orthogonal views (y-z plane; x-z plane) indicate areas of degraded gelatin with cortactin surrounded by Intβ1 rings. Right, separate channels and merged images of the selected ROI. Scale bar, 20 μm. Histograms represent the mean ± SD of the normalized degradation area percentage of cells; statistics were obtained using One-way ANOVA. **D.** Representative WB of whole cell lysates from HOFs stimulated or not with ET-1 and/or ATN161 (5 min), probed with Abs to active intβ1 and intβ1. Histograms represent the mean ± SD of densitometric analyses of proteins relative to GAPDH; statistics were obtained using One-way ANOVA.

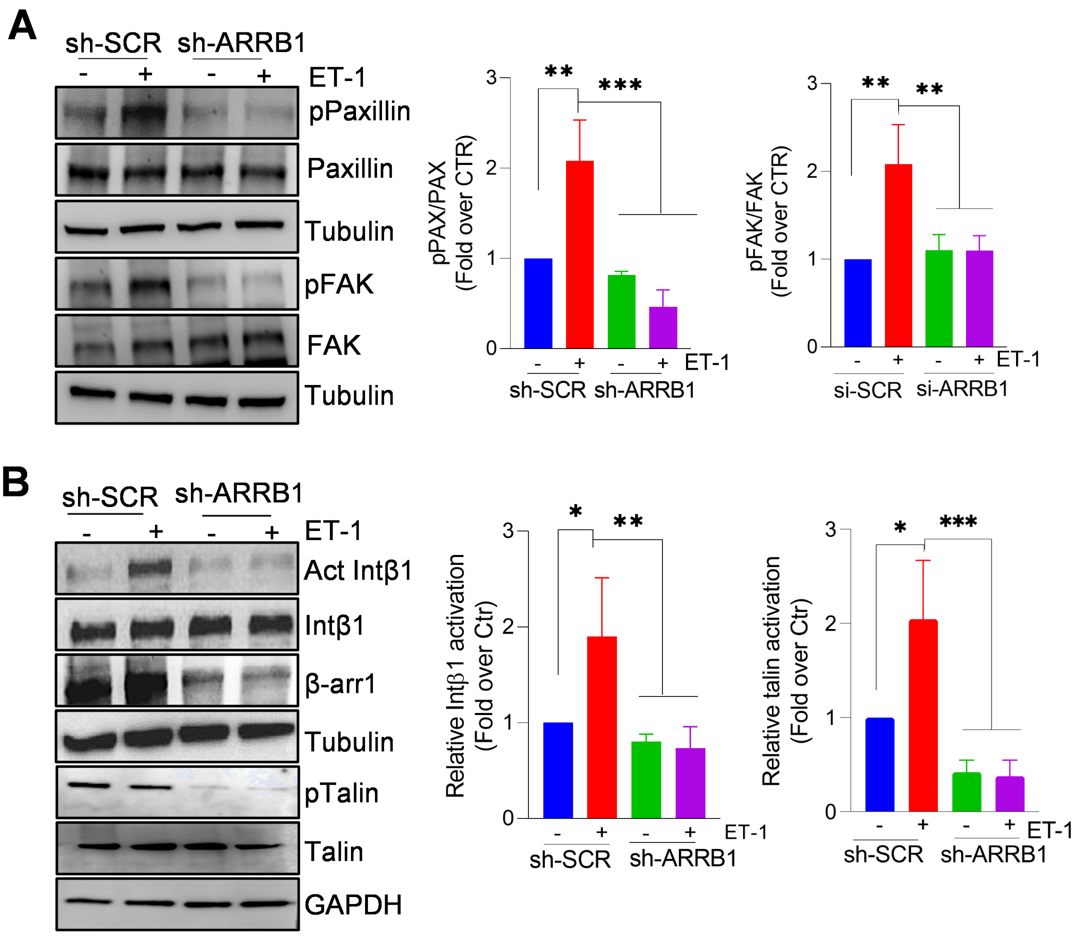

Supplementary Figure 5. ET-1/ET$_{A/B}$R axis regulates Intβ1 signaling through β-arr1. Representative WB of whole cell lysates from sh-SCR and sh-ARRB1 HOFs stimulated or not with ET-1 (5 min) and probed with **A**. Abs to pPaxillin, Paxillin, pFAK, FAK or **B.** Abs to active or total Intβ1, β-arr1, pTalin and Talin. Histograms represent the mean ± SD of densitometric analyses of proteins relative to Tubulin or GAPDH; statistics were obtained using One-way ANOVA.

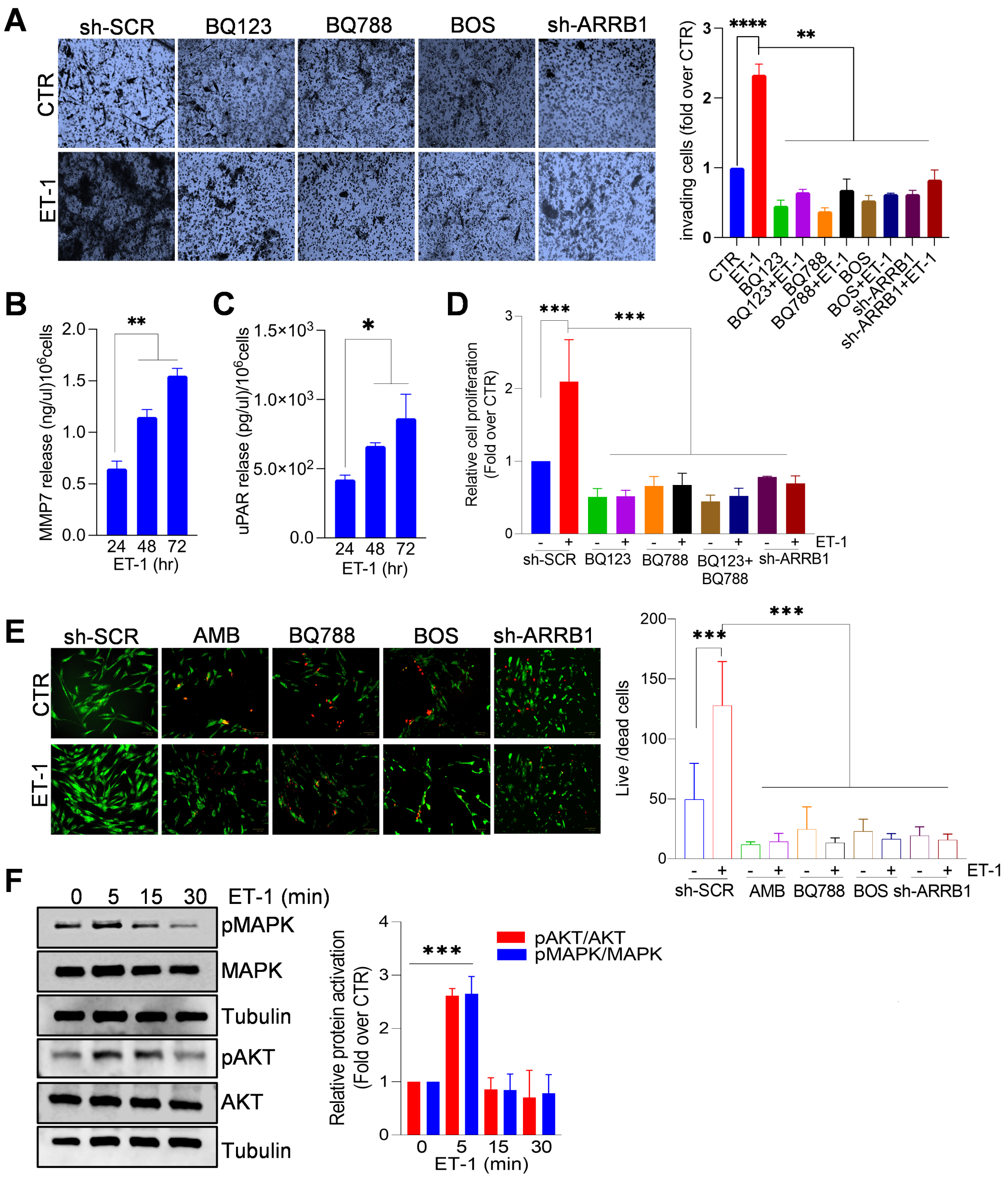

Supplementary Figure 6. ET-1 facilitates HOF invasion and protease secretion. A. Transwell invasion assays of sh-SCR- or sh-ARRB1-HOFs unstimulated or stimulated with ET-1 and/or BQ123, and/or BQ788, and/or BOS. Histograms, the mean ± SD of invaded cells (n=6); statistics were obtained using One-way ANOVA. **B.** MMP-7 or **C.** uPAR secretion evaluated by ELISA in conditioned media (CM) from HOFs untreated or treated with ET-1, at indicated times. Histograms, the mean ± SD of secreted proteins at the indicated times (n = 2); statistics were obtained using One-way ANOVA. **D.** Proliferation assay in sh-SCR or sh-ARRB1 HOFs stimulated with ET-1 and/or BQ123 and/or BQ788 and/or BOS for 48hr. Histograms, the mean ± SD of cell number (n =6), statistics were obtained using One-way ANOVA. **E)** Live-dead images of sh-SCR- or sh-ARRB1-HOFs treated with ET-1 and/or AMB and/or BQ788 and/or BOS for 48 hr. Live (green) or dead (red) cells were determined using a dual-fluorescence system. Scale bar, 100 µm. Histograms, the mean ± SD of the live/dead cell ratio (n = 2), statistics were obtained using One-way ANOVA. **F.** Representative WB of whole cell lysates from HOFs stimulated or not with ET-1 for the indicated times and probed with Abs to p-p42/44 MAPK/p42/44 MAPK and p-Akt/Akt. Histograms, the mean ± SD of densitometric analyses of proteins relative to Tubulin; statistics were obtained using One-way ANOVA.

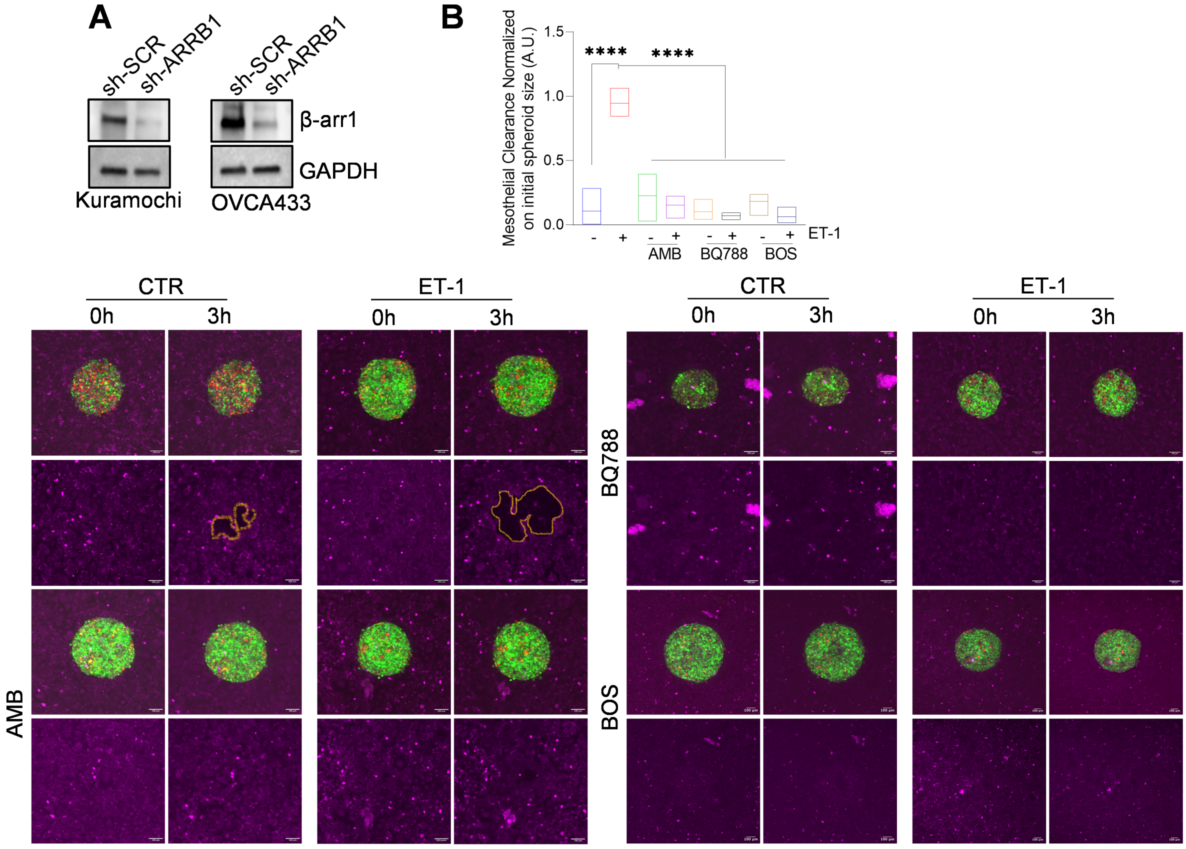

Supplementary Figure 7. A. Representative WB of whole cell lysates from sh-SCR and sh-ARRB1 OVCA433 and Kuramochi cells, probed with Ab to β-arr1. GAPDH, loading control. **B.** Images depict mesothelial clearance induced by OVCA433 (green)/HOF (red) heterotypic spheroids treated or not with ET-1 and/or AMB and/or BQ788 and/or BOS at 0- and 3-hr time points. Scale bar, 50 µm. The graph represents the ratio between the area of the "hole"/aperture in the mesothelial monolayer after 3 hr (highlighted with the yellow line) and the initial spheroid area (0 h). Statistics were obtained using One-way ANOVA.
