# Supplementary material for: The β-arrestin1/endothelin axis bolsters ovarian fibroblast-dependent invadosome activity and cancer cell metastatic potential
Source: Cell Death Dis. 2024 May 22;15(5):358. doi: 10.1038/s41419-024-06730-6 (PMC11111729; doi:10.1038/s41419-024-06730-6)
Supplement: Supplementary file 2 — Original Data [file 41419_2024_6730_MOESM2_ESM.pdf]

**Fig 1A**

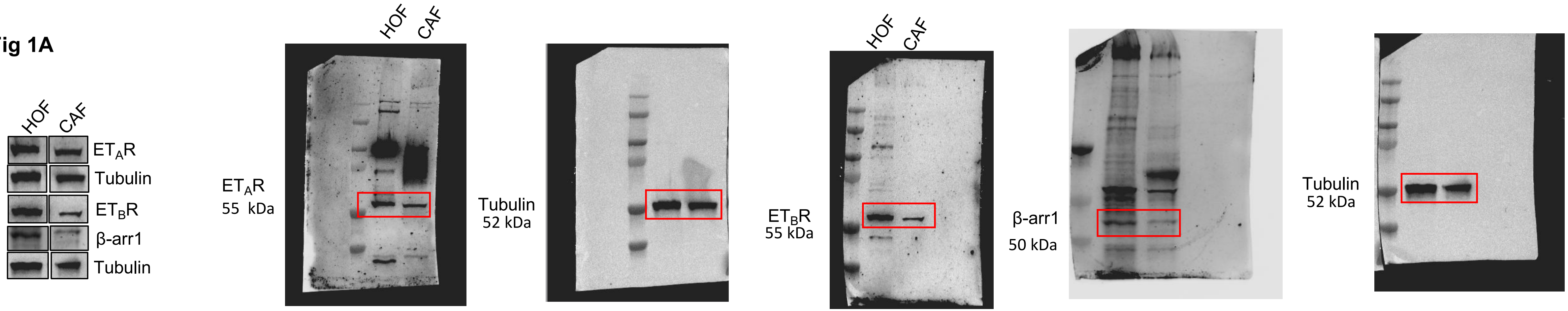
HOF
CAF
ET$_A$R
Tubulin
ET$_B$R
β-arr1
Tubulin
HOF
CAF
ET$_A$R
55 kDa
Tubulin
52 kDa
HOF
CAF
ET$_B$R
55 kDa
β-arr1
50 kDa
Tubulin
52 kDa

**Fig 2B**

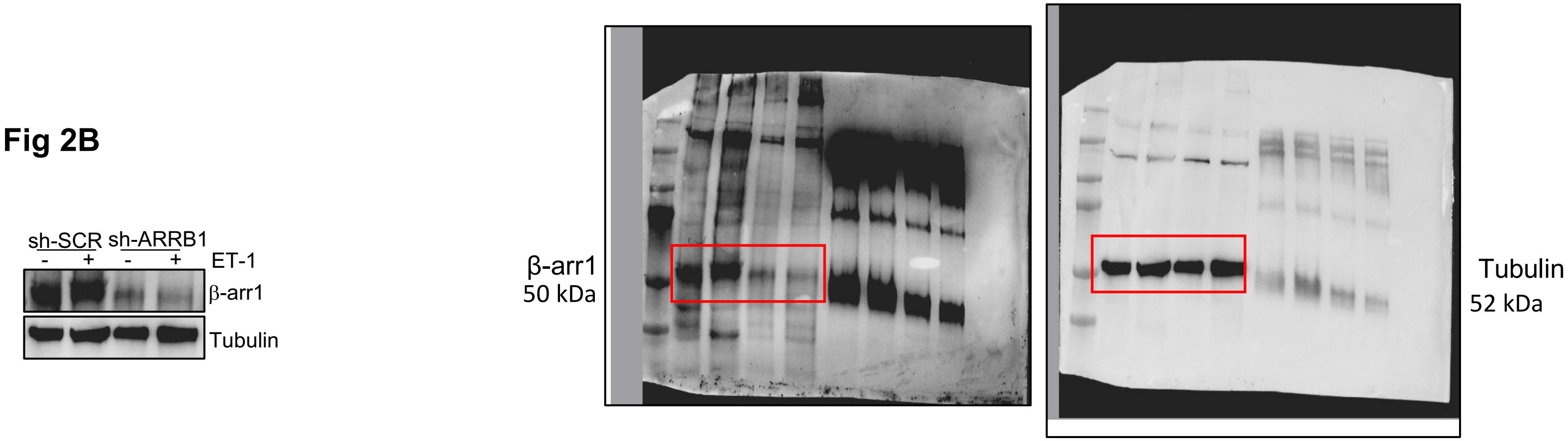
sh-SCR
sh-ARRB1
- + - +
ET-1
β-arr1
Tubulin
β-arr1
50 kDa
Tubulin
52 kDa

Fig.4A

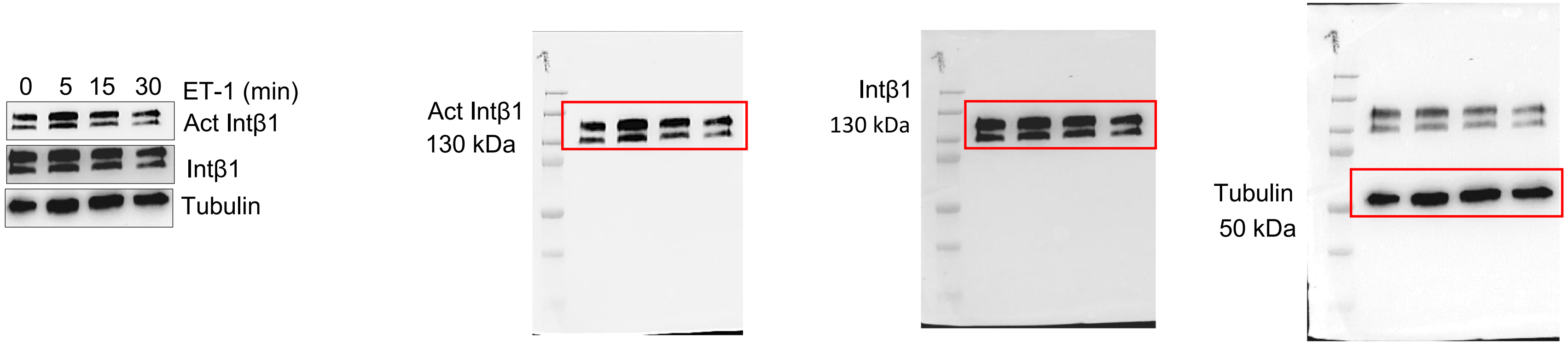
0 5 15 30 ET-1 (min)
Act Intβ1
Intβ1
Tubulin
Act Intβ1
130 kDa
Intβ1
130 kDa
Tubulin
50 kDa

Fig.4B

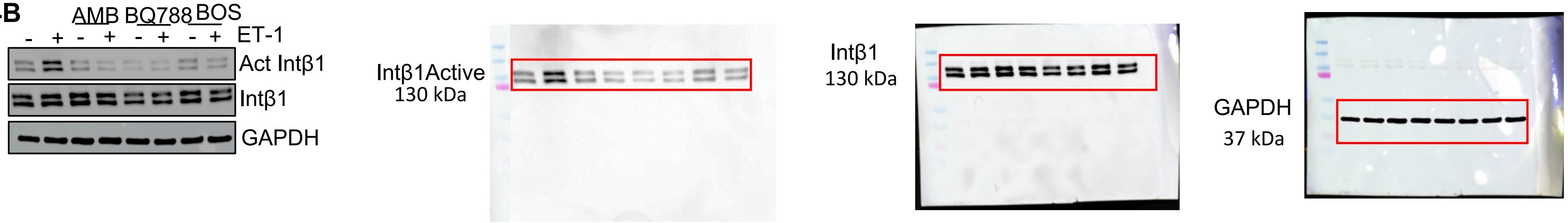
AMB BQ788 BOS
- + - + - + - + ET-1
Act Intβ1
Intβ1
GAPDH
Intβ1Active
130 kDa
Intβ1
130 kDa
GAPDH
37 kDa

**Fig 5A**

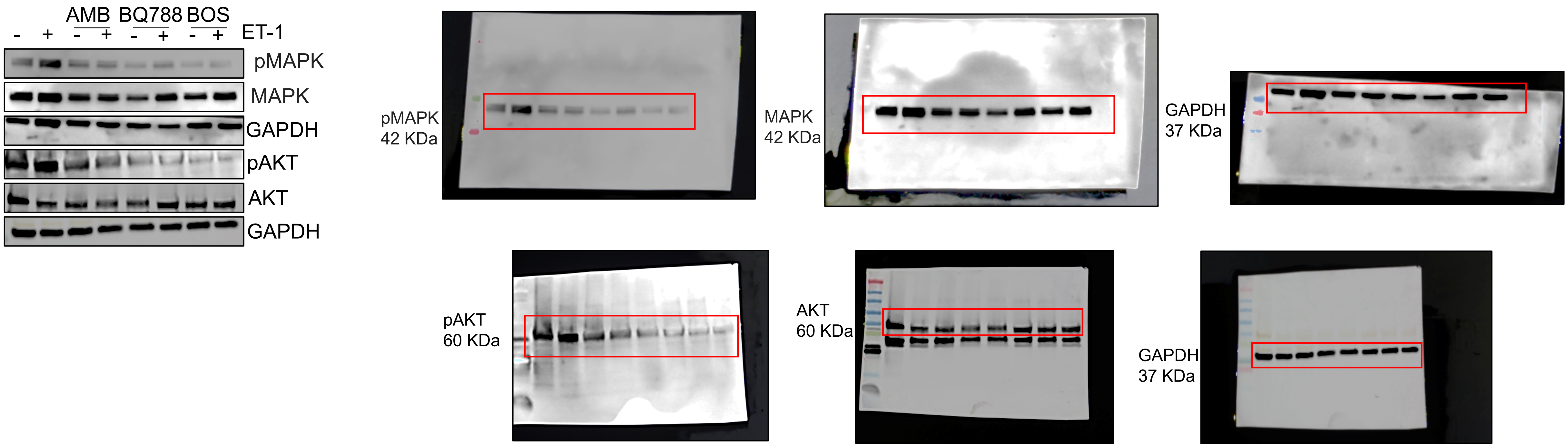
AMB
BQ788
BOS
- + - + - + - +
ET-1
pMAPK
MAPK
GAPDH
pAKT
AKT
GAPDH
pMAPK
42 KDa
MAPK
42 KDa
GAPDH
37 KDa
pAKT
60 KDa
AKT
60 KDa
GAPDH
37 KDa

Fig 7B

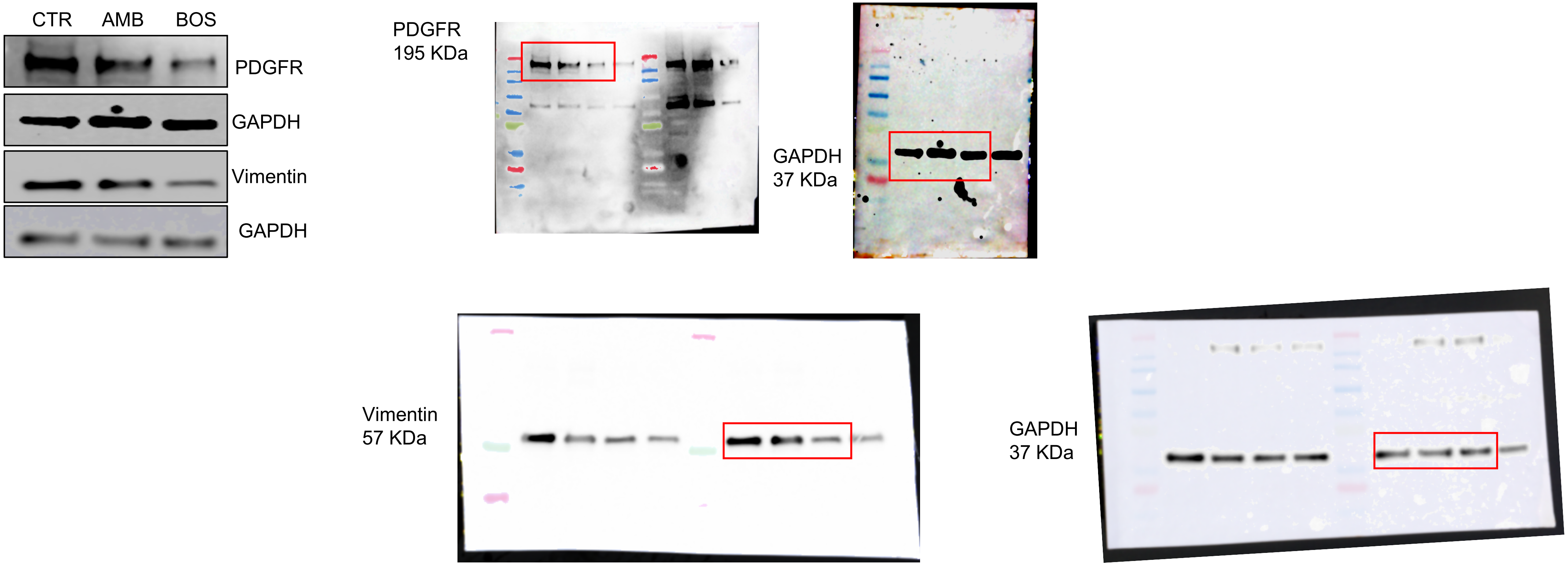
CTR
AMB
BOS
PDGFR
GAPDH
Vimentin
GAPDH
PDGFR
195 KDa
GAPDH
37 KDa
Vimentin
57 KDa
GAPDH
37 KDa

**Suppl. fig. 1A**

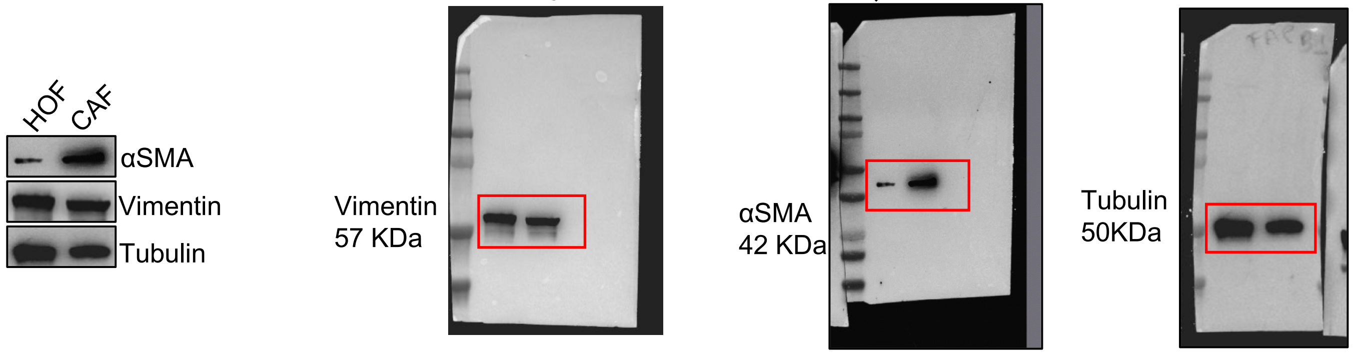


**Suppl. fig. 1C**

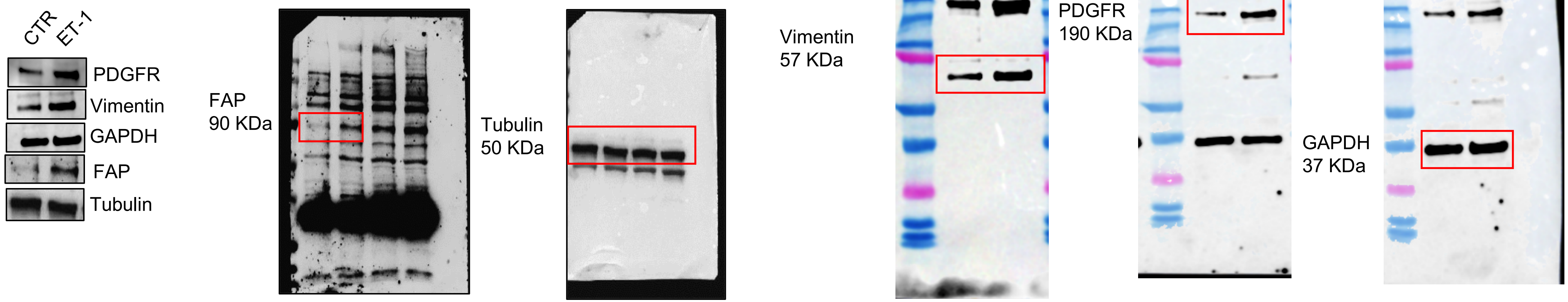


**Suppl. fig. 1D**

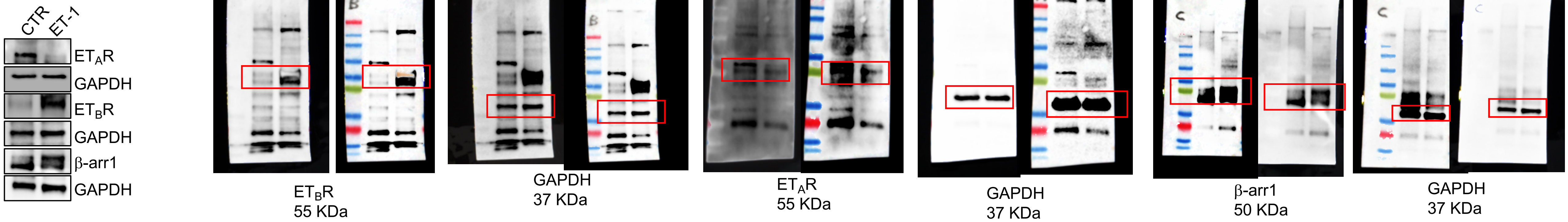

**Suppl. fig. 3B**

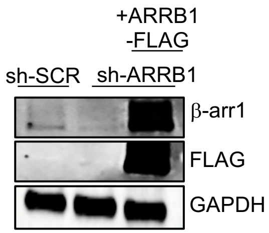


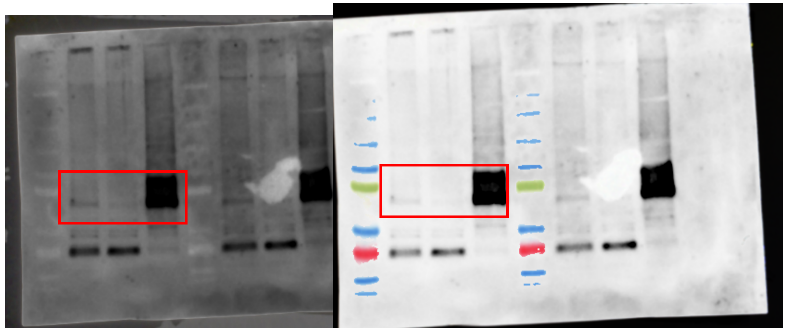

β-arr1
50 KDa

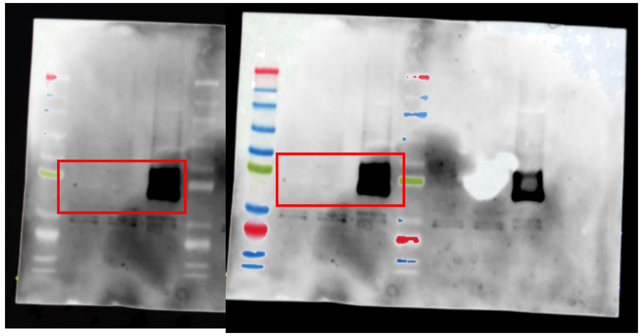

β-arr1-FLAG
50 KDa

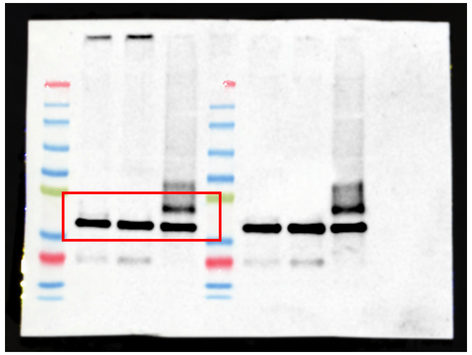

GAPDH
37 KDa

**Suppl. fig. 4A**

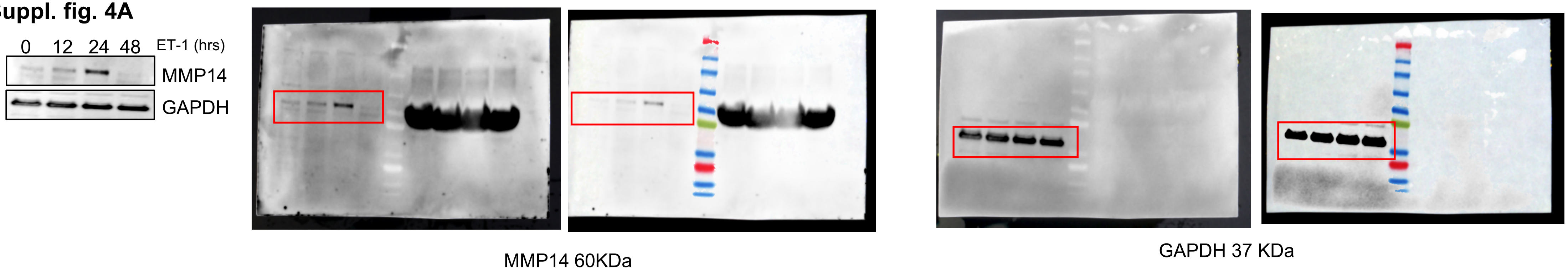


**Suppl. fig. 4B**

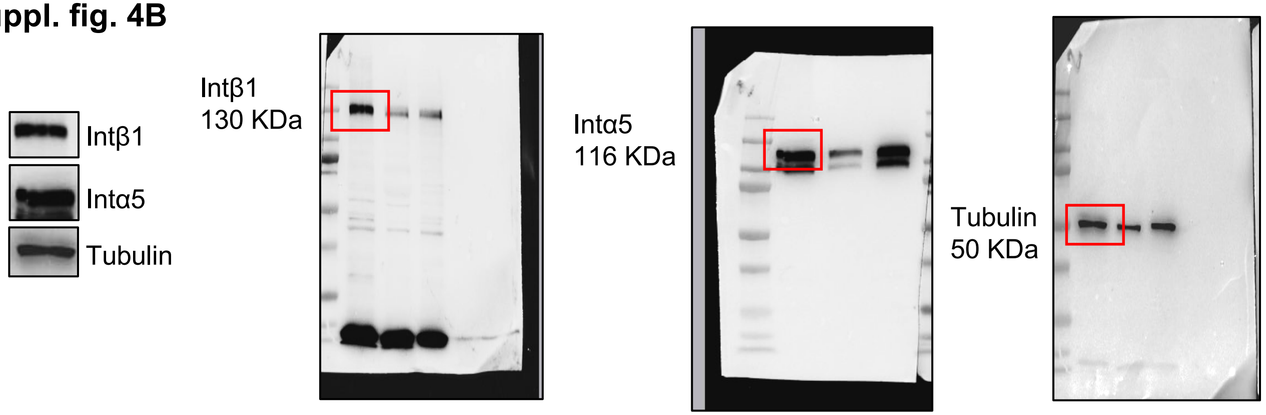


**Suppl. fig. 4D**

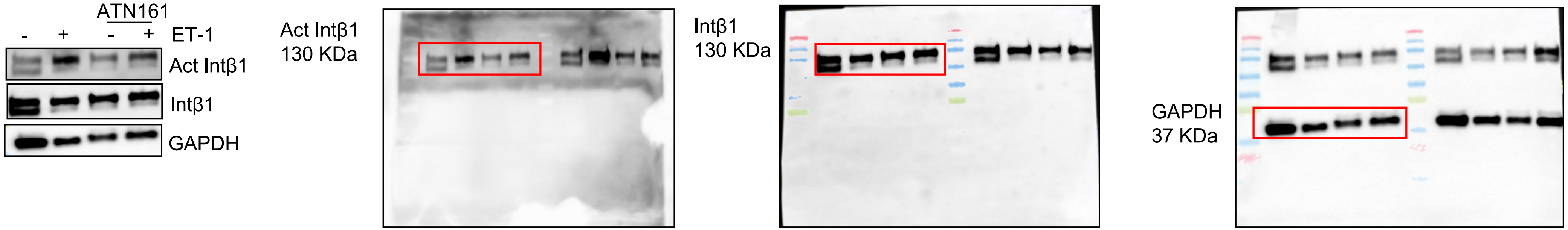

**Suppl. fig.5A**

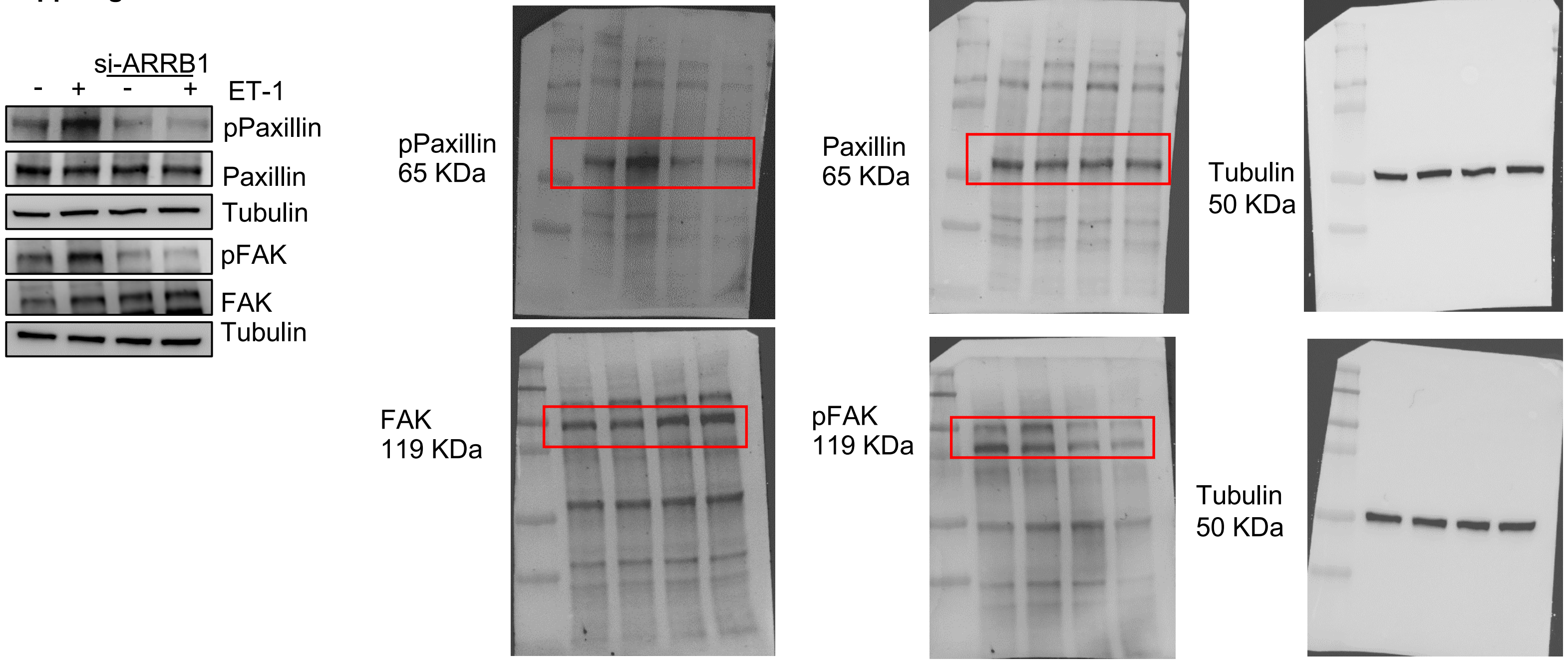


**Suppl. fig.5B**

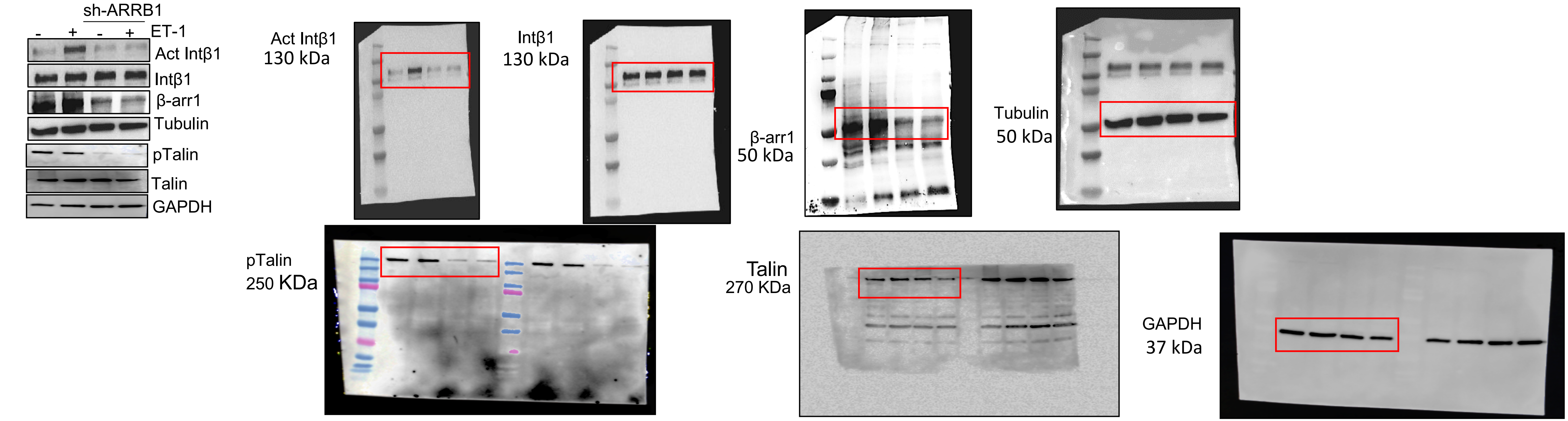

**Suppl. fig.6F**

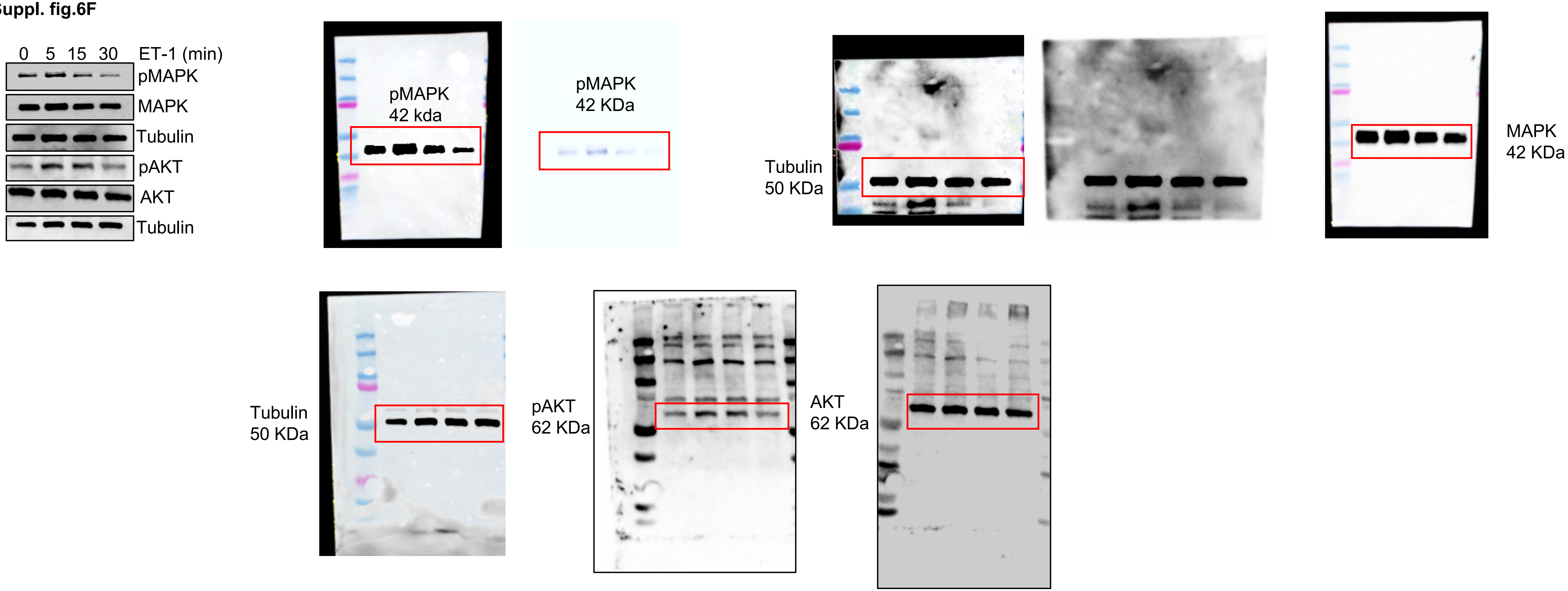
0 5 15 30 ET-1 (min)
pMAPK
MAPK
Tubulin
pAKT
AKT
Tubulin
pMAPK
42 kda
pMAPK
42 KDa
Tubulin
50 KDa
MAPK
42 KDa
Tubulin
50 KDa
pAKT
62 KDa
AKT
62 KDa

**Suppl. fig. 7A**

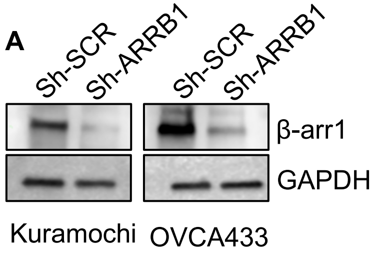
A
Sh-SCR
Sh-ARRB1
Sh-SCR
Sh-ARRB1
β-arr1
GAPDH
Kuramochi
OVCA433

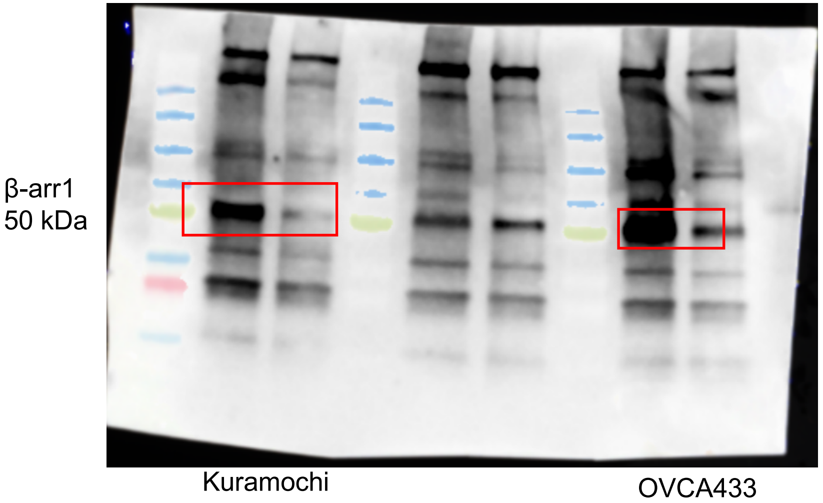
β-arr1
50 kDa
Kuramochi
OVCA433

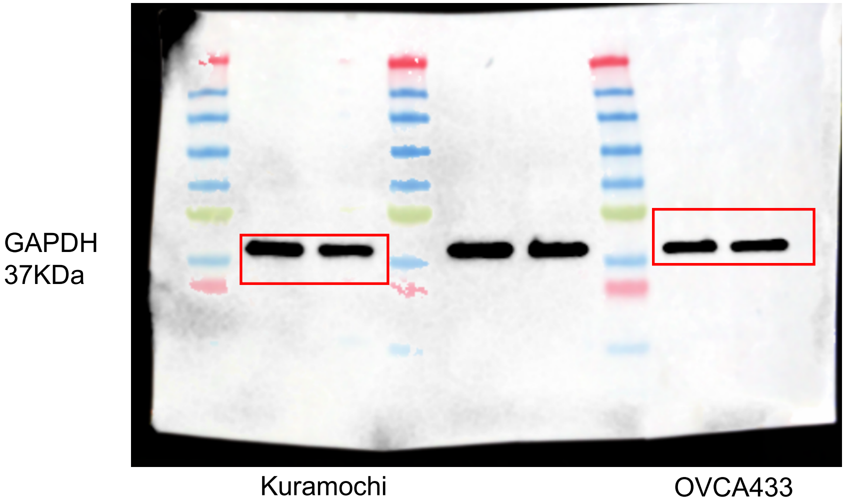
GAPDH
37KDa
Kuramochi
OVCA433
